# Granzyme K CD8⁺ T cells with tissue-resident features promote intestinal inflammation in patients with Crohn’s disease

**DOI:** 10.1038/s12276-026-01763-7

**Published:** 2026-07-06

**Authors:** Yoonho Lee, Tae-Young Kim, Yongjae Kim, Jiwon Baek, Hwan Park, Do Kyung Yoon, Sung Wook Hwang, Jong Lyul Lee, Sang Hyoung Park, Jihun Kim, Suk-Kyun Yang, Buhm Han, Mi-Na Kweon, Kyuyoung Song, Yong Sik Yoon, Byong Duk Ye, Ho-Su Lee

**Affiliations:** 1 Department of Biochemistry and Molecular Biology, Brain Korea 21 Project, Asan Medical Center, University of Ulsan College of Medicine, Seoul, Korea; 2 Mucosal Immunology Laboratory, Brain Korea 21 Project, Asan Medical Center, University of Ulsan College of Medicine, Seoul, Korea; 3 Department of Gastroenterology, Inflammatory Bowel Disease Center, Asan Medical Center, University of Ulsan College of Medicine, Seoul, Korea; 4 Division of Colon and Rectal Surgery, Department of Surgery, Inflammatory Bowel Disease Center, Asan Medical Center, University of Ulsan College of Medicine, Seoul, Korea; 5 Department of Pathology, Asan Medical Center, University of Ulsan College of Medicine, Seoul, Korea; 6 Department of Biomedical Sciences, BK21 Plus Biomedical Science Project, Seoul National University College of Medicine, Seoul, Korea

**Keywords:** Translational research, Genetics research, Gene expression

## Abstract

The role of CD8^+^ T cells in Crohn’s disease (CD) pathogenesis remains incompletely understood. This study aimed to characterize CD8^+^ T cells in CD and elucidate their potential contribution to intestinal inflammation. T cells from blood and intestinal tissues of 15 patients with CD were analyzed using single-cell RNA and T cell receptor sequencing. Spatial transcriptomics was conducted on inflamed intestinal tissues from two patients. Analysis of 41,699 CD8^+^ T cells identified distinct subsets characterized by differential granzyme expression: granzyme B (GZMB^+^) CD8^+^ T cells, predominantly in blood with high cytotoxic potential, and granzyme K (GZMK^+^) CD8^+^ T cells, enriched in intestinal tissue with lower cytotoxic potential. In the small intestine, GZMK^+^CD8^+^ T cells displayed enhanced tissue residency signatures (for example, *CXCR6*) and downregulated egress-related genes (*S1PR1*and *S1PR5*). GZMK^+^CD8^+^ T cells displayed robust interactions with myeloid cells via the CXCR3–CXCL9/10 axis, coupled with notable colocalization in the small intestine. Pharmacological inhibition of GZMK alleviated intestinal inflammation and tissue damage in a murine model of intestinal injury, supporting its role in modulating inflammatory responses. Together, these findings highlight GZMK as a potential modulator of intestinal inflammation and a candidate for further therapeutic investigation.

## CD8 T cell subsets drive inflammation in Crohn’s disease

Crohn’s disease involves complex immune dysregulation, particularly through CD8^+^ T cells, which have roles in inflammation. This study identifies a distinct subset of CD8^+^ T cells expressing granzyme K (GZMK), which are prevalent in the small intestine of patients with Crohn’s disease and exhibit unique tissue residency features. Researchers used single-cell RNA-sequencing and T cell receptor sequencing to profile these cells, revealing their transcriptional states and spatial distribution. GZMK^+^CD8^+^T cells showed lower cytotoxicity compared with GZMB^+^CD8^+^T cells and were more abundant in the small intestine, correlating with disease severity. The study highlights transcriptional programs driving tissue residency, such as increased CXCR6 expression, and regulatory networks involving AP-1 and RUNX3. These findings suggest that GZMK^+^CD8^+^ T cells contribute to local inflammation and may serve as potential therapeutic targets.

This summary was initially drafted using artificial intelligence, then revised and fact-checked by the author.

## Introduction

Crohn’s disease (CD) pathogenesis involved complex immune dysregulation, particularly T cell-mediated inflammation^1,2^. Although CD4^+^ T cell subsets in autoimmune diseases were well characterized, CD8^+^ T cell populations had been less comprehensively defined. CD8^+^ T cells played both disease-promoting and regulatory roles in inflammatory disorders, primarily through granzyme B (GZMB)-mediated cytotoxicity^3^. However, recent studies identified a prevalent CD8^+^ T cell subset in autoimmune-affected tissues expressing granzyme K (GZMK), distinct from classic cytotoxic or tissue-resident memory T cells^4–8^. The migration and retention of these cells in intestinal tissue emerged as a potentially important factor in maintaining local inflammatory responses in CD.

Therapies targeting T cell trafficking, such as vedolizumab and ozanimod, demonstrated the importance of chemokine-mediated T cell migration in CD pathogenesis^1,2^. Studies revealed associations between blood CD8^+^ T cell transcriptional patterns and CD relapse^9^, emphasizing their role in perpetuating ongoing inflammation and their potential as therapeutic targets^10,11^. However, despite advances in single-cell technologies, the specific mechanisms governing the gut-homing and tissue adaptation of potentially pathogenic GZMK-expressing CD8^+^ T cells remained poorly understood.

To address these knowledge gaps, this study used a comprehensive strategy integrating single-cell RNA-sequencing (scRNA-seq), single-cell T cell receptor sequencing (scTCR-seq), and spatial transcriptomics (ST) from both inflamed and non-inflamed regions of small intestine (SI), as well as peripheral blood, of patients with CD. We profiled CD8^+^ T cells to uncover their transcriptional states, clonal relationships, and spatial distribution within the intestinal microenvironment. In parallel, functional relevance was assessed in a murine model, supporting an in vivo role for GZMK^+^CD8^+^ T cells in intestinal injury and pathogenesis.

## Materials and methods

### Tissue collection and isolation of immune cells for scRNA-seq and scTCR-seq

We recruited 15 patients with CD for scRNA-seq and scTCR-seq and performed ST analyses on 1 of these 15 patients and 1 additional patient with CD (Supplementary Table 1). Tissue dissociation and isolation of CD3^+^ or CD45^+^ immune cells for scRNA-seq and scTCR-seq of inflamed and uninflamed SI tissues were performed, as described previously^12^. This study was conducted with ethical approval from the Institutional Review Board of Asan Medical Center (IRB no. 2019-1294) and all participants provided informed consent. All procedures were carried out in accordance with relevant guidelines and regulations.

Whole blood was collected in heparin vacutainer tubes (BD, NJ, USA), and peripheral blood mononuclear cells (PBMCs) were isolated using Ficoll-Paque® PLUS (density 1.077 ± 0.001 g/ml; GE Healthcare, UK), according to the manufacturer’s protocol. Briefly, 5 ml of blood was diluted with an equal volume of 1× PBS, layered over 5 ml Ficoll-Paque, and centrifuged at 400×*g* for 30 min at 20 °C without brake. The interface layer was collected, washed twice with 1× PBS (400×*g*, 10 min), and resuspended in CELLBANKER® freezing medium (Amsbio, UK) before storage at −80 °C. PBMCs were thawed, washed, and resuspended in PBS with 0.04% bovine serum albumin at 900–1600 cells/μl. Samples were pooled from 2 to 4 donors per batch (3 batches).

### scRNA-seq and scTCR-seq

scRNA-seq and scTCR-seq for intestinal cells were performed as described previously^12^. In brief, cells were suspended in PBS, filtered, and counted. For scRNA-seq, the 10× Genomics Chromium platform was used to generate barcoded cDNA libraries, which were then sequenced on an Illumina NovaSeq6000 system. For scTCR-seq, a nested-PCR approach was used to amplify T cell transcripts, with libraries enriched for αβ TCRs and sequenced on the same Illumina platform.

Pooled PBMCs from patients with CD were loaded on Chromium platform (10× Genomics) with a target of capturing 12,000–16,000 cells. Single-cell capturing and library construction were performed using Chromium Next GEM Single Cell 5ʹ kit v2 (10× Genomics, PN1000263) and Chromium Single Cell Human V(D)J Reagent kit (10× Genomics, PN1000252 and PN1000253). Single-cell libraries were constructed according to the manufacturer’s protocol (10× Genomics, CG000331). All libraries were sequenced on the NovaSeq 6000 platform using S2 sequencing reagent, with a target of ~600 million reads per library using dual indexing.

### scRNA-seq data analysis

Demultiplexing of raw FASTQ files, alignment to the GRCh38 reference genome, and quantification of unique molecular identifier counts were performed using CellRanger v6.1.2 (ref. ^13^). Filtered data matrices were then used for subsequent quality control and clustering analyses in Seurat v4 (ref. ^14^). High-quality cells were selected based on the following criteria: (1) cells with more than 500 genes, (2) cells with fewer than 3500 genes, and (3) cells with less than 15% mitochondrial gene expression. Potential doublets were identified and removed with DoubletFinder v2.0.3 (ref. ^15^).

For clustering, gene expression matrices were normalized by total unique molecular identifier counts per cell and log-transformed. Highly variable genes (*n* = 2000) were identified using the “vst” method. Cell cycle scores for G2/M and S phase genes were calculated and regressed during scaling. Principal component analysis was performed on the variable genes, followed by batch correction using Harmony^16^. Non-T/NK clusters were removed and clustering repeated, yielding 130,966 T/NK cells (Supplementary Table 2). CD8^+^ T cell subclustering was performed by reclustering three CD8^+^ T cell clusters. Gene signatures (Supplementary Table 3) were quantified using Seurat AddModuleScore.

### Differential expression analysis

scRNA-seq data underwent differential expression (DE) analysis using the FindMarkers function from Seurat v4. Genes were included in the DE analysis only if they were detected in at least 25% of cells within each cluster or group. Differentially expressed genes (DEGs) were defined based on an adjusted *P*-value of less than 0.05 and an absolute log2 fold change greater than 0.5 in average expression levels. Gene set enrichment analysis was carried out using the fgsea R package, and only pathways with a false discovery rate (FDR) < 0.05 were considered significant^17^.

### Differential abundance analysis

Differential abundance testing for each cell type between SI and blood was performed using the Milo algorithm^18^. Cells were assigned to partially overlapping neighborhoods in a *k*-nearest neighbor graph (*k* = 10, *d* = 30). Cell counts per neighborhood were calculated for each sample, and a generalized linear model estimated *P*-values and log2 fold changes. Neighborhoods with FDR < 0.1 were considered significant.

### Flow cytometry

Single-cell suspensions obtained by dissociation of uninflamed SI tissue were stimulated with Cell Stimulation Cocktail (eBioscience, no. 00-4970) and Brefeldin A (eBioscience, no. 00-4506-51) at 37 °C, 5% CO_2_ for 6 h. Cells were stained with Fixable Viability Dye eFluor™ 780 (eBioscience, no. 65-0865) for 30 min on ice, followed by anti-CD45 staining (BioLegend, no. 368525). Intracellular staining was performed using the True-Nuclear™ Transcription Factor Buffer Set (BioLegend, no. 424401) with antibodies against granzyme B (eBioscience, no. 12-8899-41), granzyme K (BioLegend, no. 370515), and perforin (BioLegend, no. 308112). Data were acquired on an FACS Canto II (BD) and analyzed with FlowJo v10. After excluding dead cells, CD45^+^ cells were gated and perforin expression analyzed in granzyme K^+^ and granzyme B^+^ populations.

### Gene regulatory network analysis

Gene regulatory networks involving transcription factors (TFs) and their target genes within the scRNA-seq data were calculated using the Single-Cell Regulatory Network Inference and Clustering (SCENIC) package in R^19^. Genes detected in less than 1% of total cells were filtered out. To enhance computational efficiency, 4000 cells were randomly sampled from each cluster. Co-expression modules comprising each TF and its target genes, referred to as regulons, were derived using the GENIE3 algorithm. Subsequently, the activity of each regulon in individual cells was calculated using the AUCell function across all cells.

### TCR repertoire sequencing analysis

For TCR data analysis, TCR reads from each sample were aligned to the GRCh38 Cellranger VDJ reference genome (cellranger-vdj-GRCh38-alts-ensembl-7.1.0) using Cellranger vdj software. Clonotype for individual cells was integrated into the scRNA-seq object with the scRepertoire^20^. All analyses involving TCR clonotypes were restricted to cells for which clonotype was assigned. Clonal expansion was characterized by categorizing each clonotype: single (1 clonotypes), small (1 < clonotypes ≤ 5), medium (5 < clonotypes ≤ 20), large (20 < clonotypes ≤ 50), and hyperexpanded (50 < clonotypes). Transition scores based on clonotype data for each cluster were computed using the STARTRAC R package^21^.

### Trajectory analysis

Pseudotime analysis was carried out using the monocle2 package^22^. To improve efficiency, 2500 cells were randomly sampled for each cell type, and dimensional reduction was performed with the DDRTree algorithm before ordering the cells along pseudotime. For each branch, a heatmap depicting gene expression changes over pseudotime was generated using the top 40 DEGs from GZMK^+^CD8^+^ T cells versus GZMB^+^CD8^+^ T cells in the SI. For RNA velocity analysis, spliced and unspliced RNAs were quantified with Velocyto^23^, merged across samples, and analyzed with scVelo^24^, with velocities projected onto Seurat Uniform Manifold Approximation and Projection (UMAP) coordinates.

### Receptor–ligand interaction analysis for scRNA-seq

We used the CellChat package to infer receptor–ligand interactions for each cluster in the scRNA-seq dataset, focusing on communications within the “Secreted Signaling” category of the CellChat database^25^. Among all computed cellular communications, only those detected in at least 10 cells per group were retained, and communications with a *P*-value below 0.01 were considered significant.

### ST using 10× Visium

An inflamed optimal cutting temperature-embedded frozen SI tissue block from a patient with CD was processed for 10× Visium analysis. Sections (10 μm) were mounted on Visium slides and stored at −80 °C. Methanol fixation, hematoxylin and eosin staining, and library preparation were performed according to the Visium protocol. Libraries were sequenced on NovaSeq 6000 (Illumina) targeting 120M reads per library. Reads were aligned to hg38 using Space Ranger (10× Genomics). After filtering, 3405 spots remained. Data were normalized with SCTransform, followed by clustering and UMAP. Cell type probability scores for each spot were calculated by performing integration with a public scRNA-seq dataset using Tangram^26,27^. Receptor–ligand interaction analysis of the 10× Visium dataset was performed using the stLearn method, which incorporates spatial coordinates^28^.

### ST using 10× Xenium

For 10× Xenium analysis, an optimal cutting temperature-embedded frozen tissue block from an inflamed SI of a patient with CD was sectioned and mounted on a Xenium slide. Sample preparation, including probe hybridization, ligation, and amplification, followed the manufacturer’s protocol (10× Genomics). After autofluorescence quenching and nuclear staining, slides were analyzed with the Xenium analyzer. Data were processed using XeniumRanger. A list of the 475 predesigned genes used for 10× Xenium analysis is provided in Supplementary Table 4. Cells with <10 detected genes were excluded, and downstream analyses were performed using Scanpy. Cell types were assigned by integration with a public scRNA-seq dataset using Tangram and visualized with Xenium Explorer^26,27,29^. Receptor–ligand interactions were analyzed using stLearn with spatial coordinates^28^.

### Multiplex immunohistochemistry

Multiplex immunohistochemistry (IHC) was performed by prismCDX (Gyeonggi-do, Korea). FFPE tissues were sectioned (4 μm), baked at 60 °C for ≥1 h, and stained using a Leica Bond Rx automated stainer. Slides were deparaffinized, subjected to antigen retrieval, and processed through sequential staining cycles including blocking, primary antibody incubation, horseradish peroxidase-conjugated secondary antibody incubation, and signal amplification using the Astra-TSA system. After each cycle, antibody stripping was performed before the next round. Primary antibodies against CD8 (Bio-Rad, MCA1817), CD11b (Invitrogen, PA5-79533), and granzyme K (Abcam, ab282703) were incubated for 30 min at room temperature. Fluorophores were applied according to the manufacturer’s instructions. Nuclei were counterstained with 4′,6-diamidino-2-phenylindole and slides were mounted with antifade reagent.

### Construction of mouse intestinal inflammation disease model

Six-week-old female C57BL/6 (B6) mice were purchased from OrientBio (Seongnam, Korea). Mice were housed under specific pathogen-free conditions with a 12-h light/dark cycle. Dextran sulfate sodium (DSS)-induced intestinal inflammation was established by administering 3% DSS (MP Biomedicals, cat no. 160110, CA) in drinking water for 7 days, followed by a switch to normal drinking water. D-phenylalanyl-prolyl-arginyl chloromethyl ketone (PPACK, MedChemExpress) was prepared at a concentration of 312.5 μg/ml in PBS containing 3% DMSO to inhibit GZMK activity. A dose of 62.5 μg of the inhibitor was administered intraperitoneally to each mouse every other day from day 5 to day 13. Body weight was monitored daily. On day 13, all mice were sacrificed for analysis of colon length and histological evaluation.

Colon tissues were opened longitudinally, rolled from distal to proximal, and fixed in 4% paraformaldehyde for 24 h at room temperature. The formalin-fixed paraffin-embedded intestinal tissue was sectioned and stained with hematoxylin and eosin according to standard protocols. A semi-quantitative composite scoring system previously developed with modifications was used to assess spontaneous intestinal inflammation, computed as a sum of five histological subscores^30^. The histological subscores (for each parameter: 0, absent; 1, mild; 2, moderate; 3, severe) are: mononuclear cell infiltrate (0–3), crypt hyperplasia (0–3), epithelial injury/erosion (0–3), polymorphonuclear cell infiltrates (0–3), and transmural inflammation (0, absent; 1, submucosal; 2, one focus extending into the muscularis and serosa; 3, up to five foci extending into the muscularis and serosa; 4, diffuse). The colonic inflammation was assessed by a gastrointestinal pathologist (J.K.), who was blinded to the experimental conditions of the samples.

## Results

### Distinct CD8^+^ T cell subsets in blood and SI of patients with CD

**Fig. 1:**
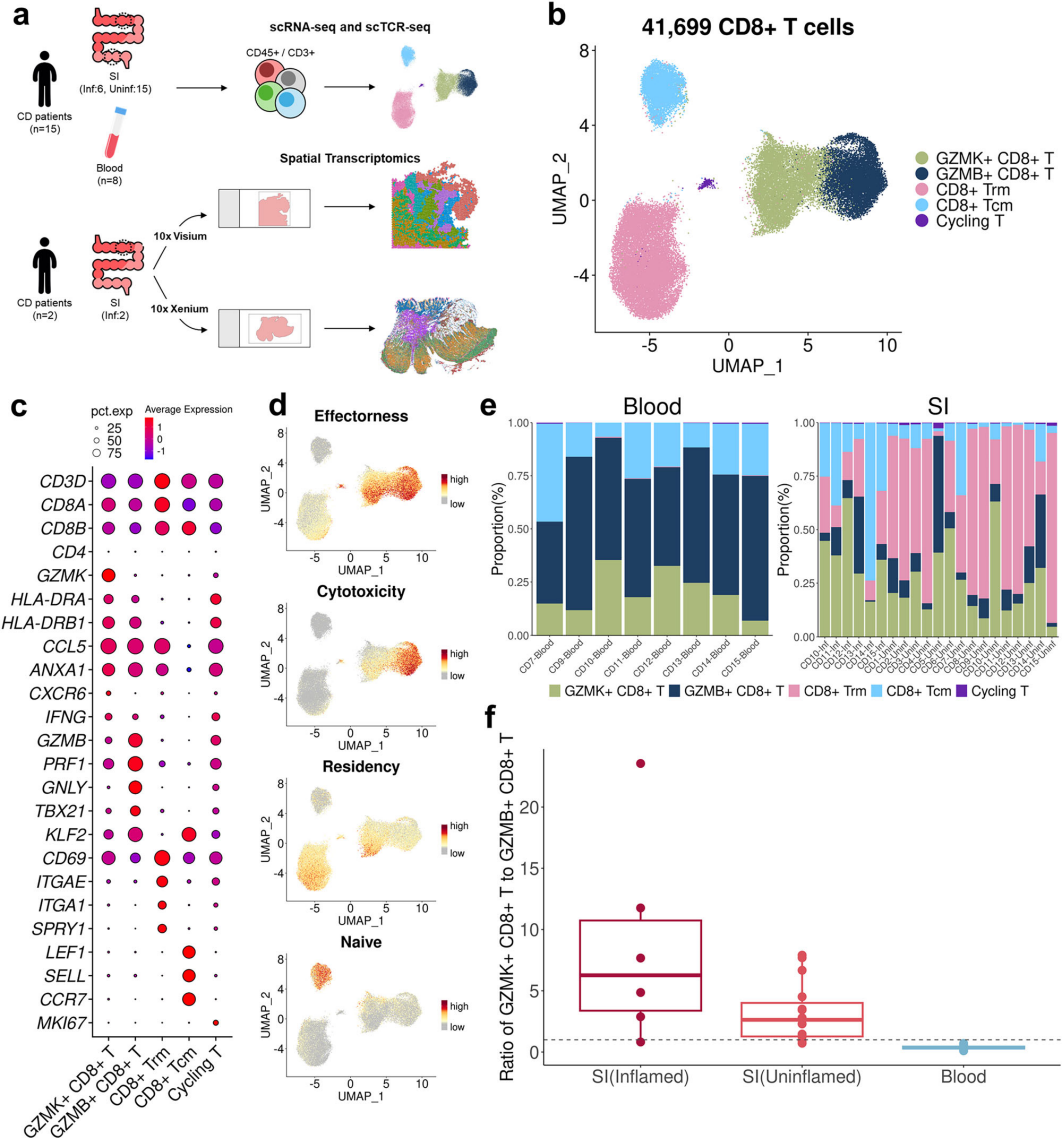
Divergent distribution patterns. **a**, A study design. **b**, UMAP (Uniform Manifold Approximation and Projection) plot of CD8^+^ T cells. **c**, Dot plot showing expression level and percentage of expressed cells for selected marker genes for CD8^+^ T cell clusters. **d**, UMAP plot depicting signature scores for naive, residency, effectorness, and cytotoxicity in individual cells. **e**, Proportions of the five major CD8^+^ T cell clusters in blood and small intestine (SI) samples. **f**, Ratios of GZMK^+^ and GZMB^+^CD8^+^ T cells within each sample of blood, inflamed SI, and uninflamed SI. CD, Crohn's disease; GZMB, granzyme B; GZMK, granzyme K; scRNA-seq, single-cell RNA-sequencing; scTCR-seq, single-cell T cell receptor sequencing.

To investigate the transcriptional signatures of CD8^+^ T cell populations in CD, we performed analysis on CD8^+^ T cells extracted from the entire T/NK cell pool (Fig. 1a,b and Supplementary Fig. 1a). This analysis revealed five distinct clusters within the CD8^+^ T cell population (Fig. 1c,d): two effector clusters characterized by high expression of *GZMK* or *GZMB* (GZMK^+^CD8^+^ T and GZMB^+^CD8^+^ T), a tissue-resident memory T cell cluster expressing tissue residency markers such as *ITGAE* and *ITGA1* (CD8^+^ Trm), a central memory T cell cluster expressing *CCR7* and *SELL* (CD8^+^ Tcm), and a proliferative T cell cluster displaying high *MKI67* expression (Cycling T).

Among the identified clusters, GZMK^+^CD8^+^ T cells and GZMB^+^CD8^+^ T cells exhibited pronounced effector phenotypes but displayed distinct functional characteristics and tissue distribution patterns (Fig. 1d,e and Supplementary Fig. 1b,c). Functionally, GZMK^+^CD8^+^ T cells demonstrated markedly lower cytotoxicity compared with GZMB^+^CD8^+^ T cells, consistent with previous studies on GZMK^+^CD8^+^ T cells and supported by reduced perforin expression at the protein level^4,5,31^ (Fig. 1d and Supplementary Fig. 1d,e). Regarding tissue distribution, we observed substantial differences in CD8^+^ T cell subset composition between blood and SI (Fig. 1e and Supplementary Fig. 1c). GZMK^+^CD8^+^ T cells were more abundant in the SI compared with blood, whereas GZMB^+^CD8^+^ T cells showed predominant enrichment in blood. This differential distribution resulted in an increased ratio of GZMK^+^CD8^+^ T cells to GZMB^+^CD8^+^ T cells in SI compared with blood (Fig. 1f). GZMK^+^ CD8^+^ T cells showed preferential enrichment in the SI of patients with CD, coupled with lower cytotoxic potential. This pattern had shared similarities with observations in tissues affected by other autoimmune and inflammatory diseases^4,6–8,31,32^, suggesting a potential role for these cells in tissue-specific inflammatory responses in CD.

### Association between GZMK^+^CD8^+^ T cell abundance and clinical disease severity

To assess whether GZMK^+^CD8^+^ T cell accumulation in the SI was associated with clinical disease severity, we performed correlation analyses between the proportion of GZMK^+^CD8^+^ T cells and established CD severity markers, including clinical disease activity index, hemoglobin, albumin, and fecal calprotectin (Supplementary Fig. 1f). In the SI, the proportion of GZMK^+^CD8^+^ T cells showed a significant negative correlation with hemoglobin levels (Spearman *R* = −0.58, *P* = 0.02), indicating that patients with higher tissue infiltration of GZMK^+^CD8^+^ T cells exhibited more severe anemia. We also observed trends toward positive correlations with clinical disease activity index (*R* = 0.38, *P* = 0.20) and fecal calprotectin (*R* = 0.50, *P* = 0.20), although these associations did not reach statistical significance, likely reflecting the limited sample size. These findings suggested that tissue-infiltrating GZMK^+^CD8^+^ T cells in the SI were associated with local disease severity, warranting further mechanistic investigation of their role in intestinal inflammation and tissue damage.

### Transcriptional signatures driving tissue residency in GZMK^+^CD8^+^ T cells

**Fig. 2:**
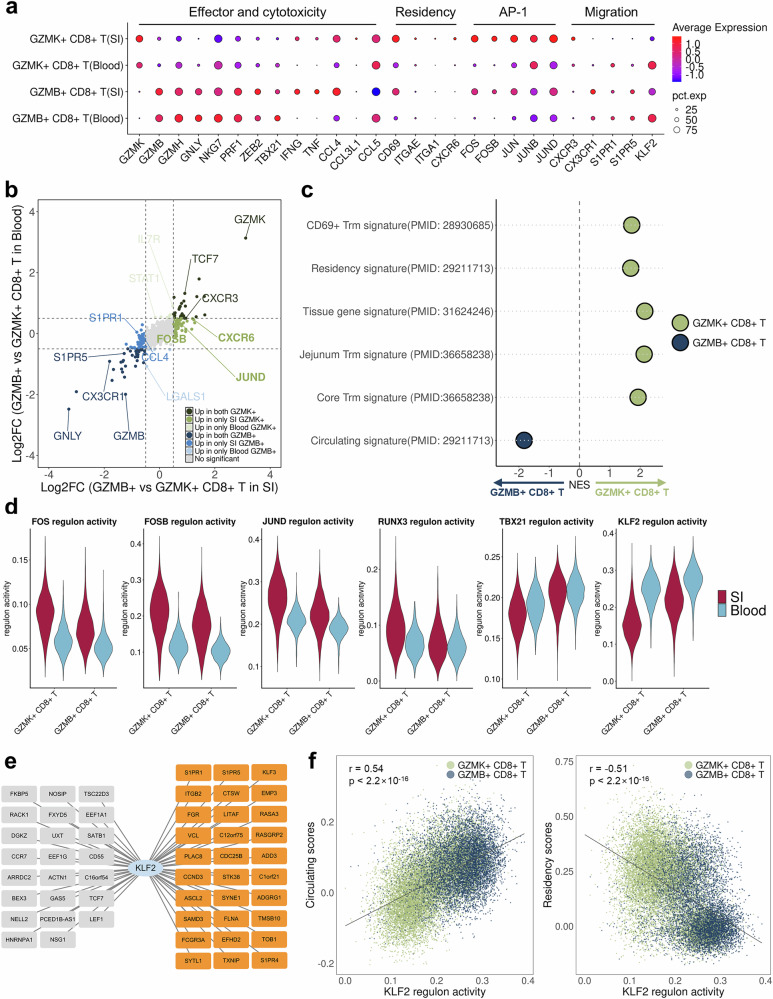
Distinct transcriptional programs. **a**, Dot plot showing the expression levels and percentage of expressed cells for signature genes in GZMK^+^ and GZMB^+^CD8^+^ T cells from the blood and small intestine (SI). **b**, The results of DE analysis between GZMK^+^ and GZMB^+^CD8^+^ T cells in the blood and SI. **c**, Dot plot depicting the results of gene set enrichment analysis for previously defined residency or circulating signatures using DEGs between GZMK^+^ and GZMB^+^CD8^+^ T cells in SI. **d**, Violin plots illustrating the regulon scores of GZMK^+^ and GZMB^+^CD8^+^ T cells in the blood and SI. **e**, Network visualization of *KLF2* and its predicted target genes from the gene regulatory network analysis, with orange-colored genes indicating those significantly downregulated in GZMK^+^CD8^+^ T cells compared with GZMB^+^CD8^+^ T cells in the gene level. **f**, Scatter plots displaying the correlation between KLF2 regulon activities and circulating or residency scores in GZMK^+^ and GZMB^+^CD8^+^ T cells from the SI. FC, fold change; GZMB, granzyme B; GZMK, granzyme K.

To investigate the transcriptional signatures driving the enrichment of GZMK^+^CD8^+^ T cells in the SI, we conducted DE analysis between GZMK^+^CD8^+^ T cells and GZMB^+^CD8^+^ T cells across inflamed and uninflamed SI samples, as well as blood compartments (Supplementary Fig. 2a–c). Our analysis revealed distinct transcriptional programs associated with tissue residency and egression in the SI (Fig. 2a–c and Supplementary Fig. 2d). SI GZMK^+^CD8^+^ T cells showed increased expression of *CXCR6*, a chemokine receptor implicated in long-term tissue residence and Trm development^33,34^ (Fig. 2a,b and Supplementary Fig. 2b,c). Additionally, SI GZMK^+^CD8^+^ T cells exhibited exclusive upregulation of AP-1 transcription factors, including *FOS*, *FOSB*, and *JUN*. These factors have essential roles in the tissue adaptation^35–37^, suggesting their importance in establishing the tissue-resident phenotype of GZMK^+^CD8^+^ T cells in the SI (Fig. 2a,b and Supplementary Fig. 2b,c). By contrast, GZMB^+^CD8^+^ T cells displayed elevated expression of *S1PR5* and *CX3CR1*, which are associated with tissue egress and blood-confined CD8^+^ T cells, respectively^11,38^ (Fig. 2a,b and Supplementary Fig. 2b,c). This differential gene expression pattern suggested distinct migratory and residency properties of these two CD8^+^ T cell subsets. GZMK^+^CD8^+^ T cells exhibited characteristics that may favor tissue retention, whereas GZMB^+^CD8^+^ T cells showed features potentially associated with circulation and tissue egress.

Gene Set Enrichment Analysis further corroborated these observations, revealing a pronounced enrichment of tissue residency signatures in GZMK^+^CD8^+^ T cells within the SI and substantial enrichment of circulating signatures in GZMB^+^CD8^+^ T cells^39–42^ (Fig. 2c and Supplementary Table 3). Notably, GZMK^+^CD8^+^ T cells from the blood still retained a pronounced circulating signature (Supplementary Fig. 2d), indicating that the enhanced tissue residency of GZMK^+^CD8^+^ T cells was largely confined to the SI microenvironment. These findings indicated a potential transcriptional basis for the observed differences in tissue localization between GZMK^+^ and GZMB^+^CD8^+^ T cells.

### Gene regulatory network enhancing the transcriptional dynamics of GZMK^+^CD8^+^ T cells

To elucidate the regulatory networks driving the distinct expression profiles between GZMK^+^ and GZMB^+^CD8^+^ T cells, we used SCENIC analysis to compare the activity of TF regulons^19^ (Supplementary Fig. 3). In the SI, GZMK^+^CD8^+^ T cells exhibited higher regulon activity for various AP-1 TFs compared with GZMB^+^CD8^+^ T cells, reflecting the gene-level expression differences observed between these two clusters (Fig. 2d). GZMK^+^CD8^+^ T cells in the SI also exhibited elevated RUNX3 regulon activity. This increased RUNX3 activity was linked to tissue residency and formation of memory CD8^+^ T cells^42,43^, potentially explaining the long-term retention of GZMK^+^CD8^+^ T cells in the SI. Notably, GZMK^+^CD8^+^ T cells in the SI displayed markedly lower KLF2 regulon activity compared with other groups (Fig. 2d,e). The KLF2 regulon included *S1PR1* and *S1PR5* as target genes^38^, both of which showed significantly reduced expression in SI GZMK^+^CD8^+^ T cells. Considering that *S1PR1* and *S1PR5* promoted T cell egress from tissues, the diminished KLF2 regulon activity and reduced expression of these genes in SI GZMK^+^CD8^+^ T cells likely served as key regulatory factors decreasing their circulating capacity (Fig. 2f). This complex transcriptional landscape, characterized by upregulation of tissue residency factors, enhanced AP-1 and RUNX3 activity, and suppression of egress-promoting genes, likely contributed to the preferential accumulation and retention of GZMK^+^CD8^+^ T cells in the SI of patients with CD.

### Developmental pathways and clonal dynamics of GZMK^+^CD8^+^ T cells

**Fig. 3:**
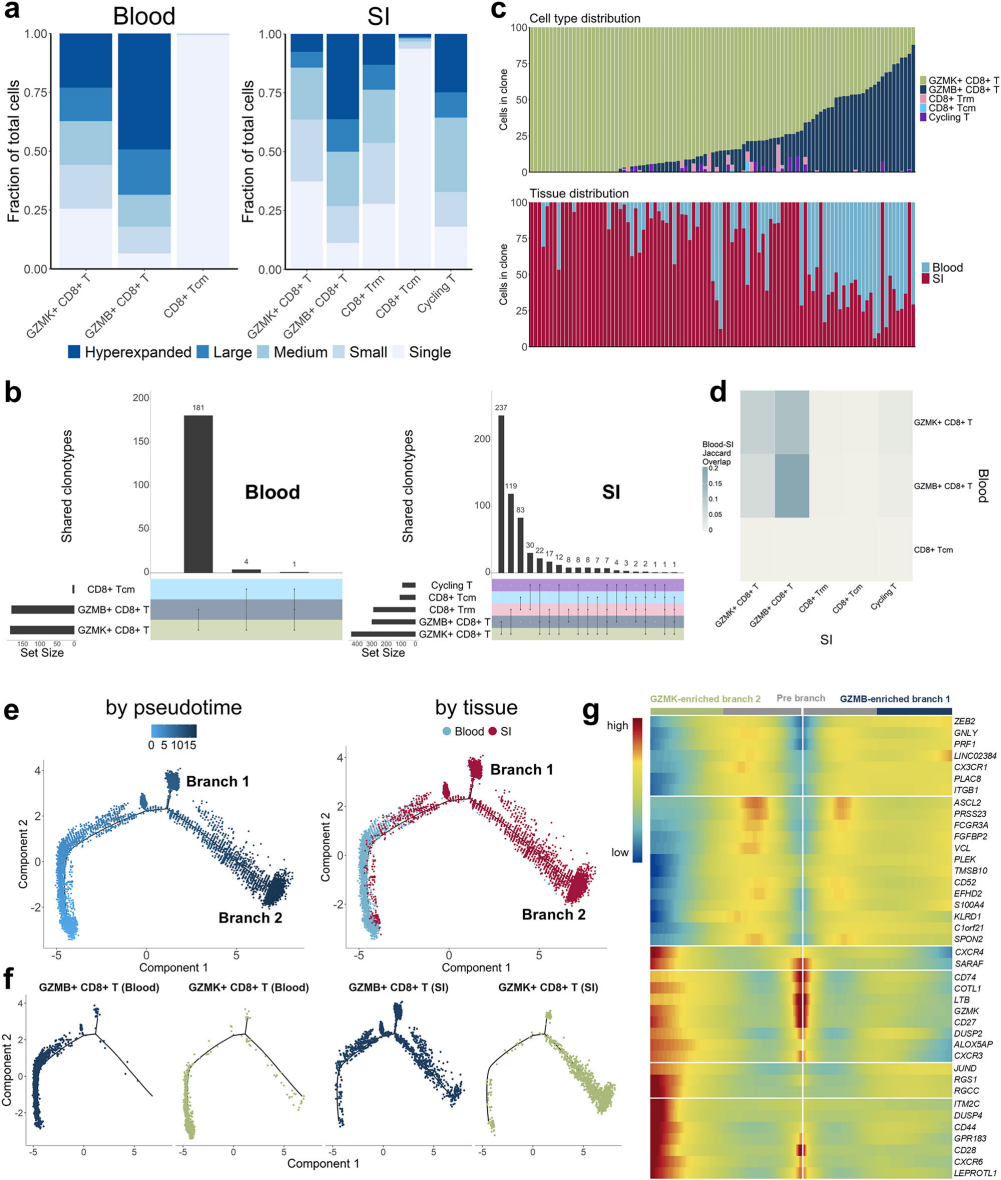
Clonal analysis and pseudotime trajectories. **a**, Bar plots showing the T cell receptor clonality of each CD8^+^ T cell cluster. **b**, UpSet plots representing the number of shared clonotypes between cells of different CD8^+^ T cell clusters. Black circles connected by a black line mean that shared clonotypes exist in the connected clusters. The number above the top bar indicates the number of shared clonotypes between the clusters. The bar on the left of each cluster represents the number of clonotypes shared with at least one other cluster. CD8^+^ T cell clusters in blood with fewer than 100 cells were excluded from the analysis. **c**, Cell type (top) and tissue (bottom) distributions of the top 100 clonotypes in small intestine (SI) GZMK^+^CD8^+^ T cells. **d**, T cell receptor repertoire similarity between blood and SI CD8^+^ T cell clusters, calculated using the weighted Jaccard index. CD8^+^ T cell clusters in blood with fewer than 100 cells were excluded from the analysis. Monocle trajectory inference across pseudotime, tissue compartments (part **e**) and cell types composed of GZMK^+^ and GZMB^+^CD8^+^ T cells from blood and SI (part **f**). **g**, Heatmap depicting gene expression changes along two distinct pseudotime branches. GZMB, granzyme B; GZMK, granzyme K.

To elucidate the developmental trajectories and clonal relationships of GZMK^+^CD8^+^ T cells, we conducted analyses of clonal expansion, shared clonotypes, and pseudotime trajectories. Clonal expansion analysis demonstrated that GZMB^+^CD8^+^ T cells exhibited higher clonality than GZMK^+^CD8^+^ T cells in both blood and SI (Fig. 3a). Despite this difference, a substantial number of clonotypes were shared between GZMK^+^ and GZMB^+^CD8^+^ T cells in both compartments, suggesting active transitions between these subsets (Fig. 3b,c). Notably, GZMK^+^CD8^+^ T cells in the SI showed a stronger clonal relationship with CD8^+^ Trm cells compared with GZMB^+^CD8^+^ T cells. Specifically, GZMK^+^CD8^+^ T cells shared 119 clonotypes with CD8^+^ Trm cells, whereas GZMB^+^CD8^+^ T cells shared only 8 clonotypes in the SI (Fig. 3b). This finding indicated a closer developmental link between GZMK^+^CD8^+^ T cells and CD8^+^ Trm cells in the SI. Moreover, examination of the degree of clonal overlap across different tissues revealed that GZMB^+^CD8^+^ T cells displayed a greater number of shared clonotypes than GZMK^+^CD8^+^ T cells, further reinforcing the notion that SI GZMK^+^CD8^+^ T cells have diminished circulating capacity (Fig. 3d).

To further elucidate the developmental pathways of these subsets, we performed pseudotime trajectory analysis, separating GZMK^+^ and GZMB^+^CD8^+^ T cells by tissue (Fig. 3e,f). This analysis revealed two discrete developmental branches originating from blood-derived CD8^+^ T cells. The SI GZMB^+^CD8^+^ T-enriched branch maintained expression of cytotoxicity-related genes such as *GNLY* and *PRF1* along the trajectory (Fig. 3g). By contrast, the SI GZMK^+^ CD8^+^ T-enriched branch showed a gradual downregulation of cytotoxicity-associated genes and a progressive increase in the expression of *CXCR6*. The unique developmental trajectory of GZMK^+^CD8^+^ T cells, characterized by reduced cytotoxicity and increased expression of tissue residency-associated factors, suggested their potential role in establishing a long-term tissue-resident population that contributed to local immune responses in CD.

### Characterization of GZMK^+^GZMB^+^ double-positive CD8^+^ T cells

The substantial clonal overlap between GZMK^+^ and GZMB^+^CD8^+^ T cells in the SI prompted us to characterize the GZMK^+^GZMB^+^ double-positive population, which constituted ~25% of GZMK-expressing cells (Fig. 1c). We subdivided the GZMK⁺CD8⁺ T cell population into GZMK⁺GZMB⁺ cells and GZMK-only cells based on GZMB expression. These double-positive cells exhibited an intermediate transcriptional profile between GZMK-only and GZMB^+^CD8^+^populations. They showed elevated expression of cytotoxicity-related genes (*GZMB*, *PRF1*, *NKG7*, and *GNLY*) compared with GZMK-only cells, while displaying reduced expression of AP-1 transcription factors (*FOS*, *FOSB*, *JUN*, and *JUNB*), consistent with a transitional state within the GZMK^+^ population (Supplementary Fig. 4a). Module score analysis revealed that GZMK^+^GZMB^+^ cells exhibited high tissue residency signatures comparable to GZMK-only cells and substantially higher than GZMB^+^CD8^+^ T cells, while their circulation signatures remained low, similar to GZMK-only cells (Supplementary Fig. 4b). TCR clonotype analysis revealed that GZMK^+^GZMB^+^ cells shared substantial clonal overlap with both GZMK-only and GZMB^+^CD8^+^ T cells in the SI, indicating active transitions between these states (Supplementary Fig. 4c). Transition score analysis, which quantifies the extent of shared TCR clonotypes between cell populations, revealed that GZMK^+^GZMB^+^ cells showed moderate transition relationships with CD8^+^ Trm cells (transition score: 0.021) and intermediate between the CD8^+^ Trm transition scores observed for GZMK-only cells (0.048) and GZMB^+^CD8^+^ T cells (0.012) (Supplementary Fig. 4d). These findings indicated that GZMK^+^GZMB^+^ cells represent a transitional CD8^+^ T cell state characterized by intermediate cytotoxic features and preserved tissue residency.

### Functional heterogeneity of GZMK^+^CD8^+^ T cells

The observation that SI GZMK^+^CD8^+^ T cells exhibited strong clonal relationships with both GZMB^+^CD8^+^ T cells and CD8^+^ Trm cells — two subsets with contrasting characteristics — led us to investigate the heterogeneity within the GZMK^+^CD8^+^ T population (Fig. 3b). GZMB^+^ CD8^+^ T cells displayed a pronounced circulating signature, whereas CD8^+^ Trm cells were characterized by tissue residency. Using ROGUE^44^, an entropy-based method, we quantified the transcriptional heterogeneity. This analysis demonstrated that GZMK^+^CD8^+^ T cells exhibited substantial transcriptional heterogeneity than GZMB^+^CD8^+^ T cells (Supplementary Fig. 5a). Building on this observation, we performed a subclustering analysis of GZMK^+^CD8^+^ T cells, which uncovered three functionally distinct subclusters, each with unique gene expression profiles and potential roles in the inflammatory process (Supplementary Fig. 5b,c).

The cytotoxic GZMK^+^ subcluster exhibited elevated levels of conventional cytotoxic T lymphocyte markers, including *NKG7*, *GNLY*, and *PRF1*. This gene expression profile indicates a more pronounced cytotoxic potential, suggesting a possible role in direct tissue damage during inflammatory conditions. A stem-like GZMK^+^ subcluster was defined by the selective expression of genes associated with T cell stemness, such as *TCF7* and *CD27*. This subpopulation likely represents a reservoir of less-differentiated cells with the potential for self-renewal and differentiation into other effector subtypes. The presence of this stem-like subcluster may contribute to the persistence of inflammation in CD, aligning with recent findings from studies on ulcerative colitis^45^. The third subcluster, termed Trm-like GZMK^+^, was characterized by the expression of *CXCR6*, along with various AP-1 TFs. This expression pattern suggests a phenotype adapted for long-term tissue residence and local immune surveillance. Notably, the Trm-like GZMK^+^ subcluster was preferentially expanded in the SI and exhibited terminally differentiated cell states (Supplementary Fig. 5d,e). Furthermore, this subcluster shared the greatest number of clonotypes with CD8^+^ Trm cells and displayed a unique functional profile characterized by elevated expression of pro-inflammatory cytokines, including *IFNG* and *TNF* (Supplementary Fig. 5f,g). These findings collectively supported a model in which a portion of terminally differentiated Trm-like GZMK^+^CD8^+^ T cells may be linked with CD8^+^ Trm in the SI of patients with CD.

To infer the gene regulatory networks of GZMK^+^CD8^+^ T subclusters, we performed regulatory network analysis (Supplementary Fig. 5h). Stem-like GZMK^+^ subcluster was enriched for LEF1 and TCF7 regulons, consistent with its expression of T cell stemness genes. By contrast, the Trm-like GZMK^+^ subcluster appeared to be regulated by *EGR2*, which in turn could regulate the transcription of AP-1 TFs, including *FOS*, *FOSB*, and *JUN*, by incorporating them among its target genes (Supplementary Fig. 5i). Additionally, a study in murine systems has shown that *EGR2* negatively regulates the cytotoxic T lymphocyte-like phenotype in CD8^+^ T cells, particularly the expression of *GZMB* and *TBX21*(ref.^46^). These findings highlighted *EGR2* as a pivotal regulator that might shape the functional divergence of Trm-like GZMK^+^CD8^+^ T cells in local inflammation.

### Crosstalk between GZMK^+^CD8^+^ T cells and myeloid cells in SI microenvironment

**Fig. 4:**
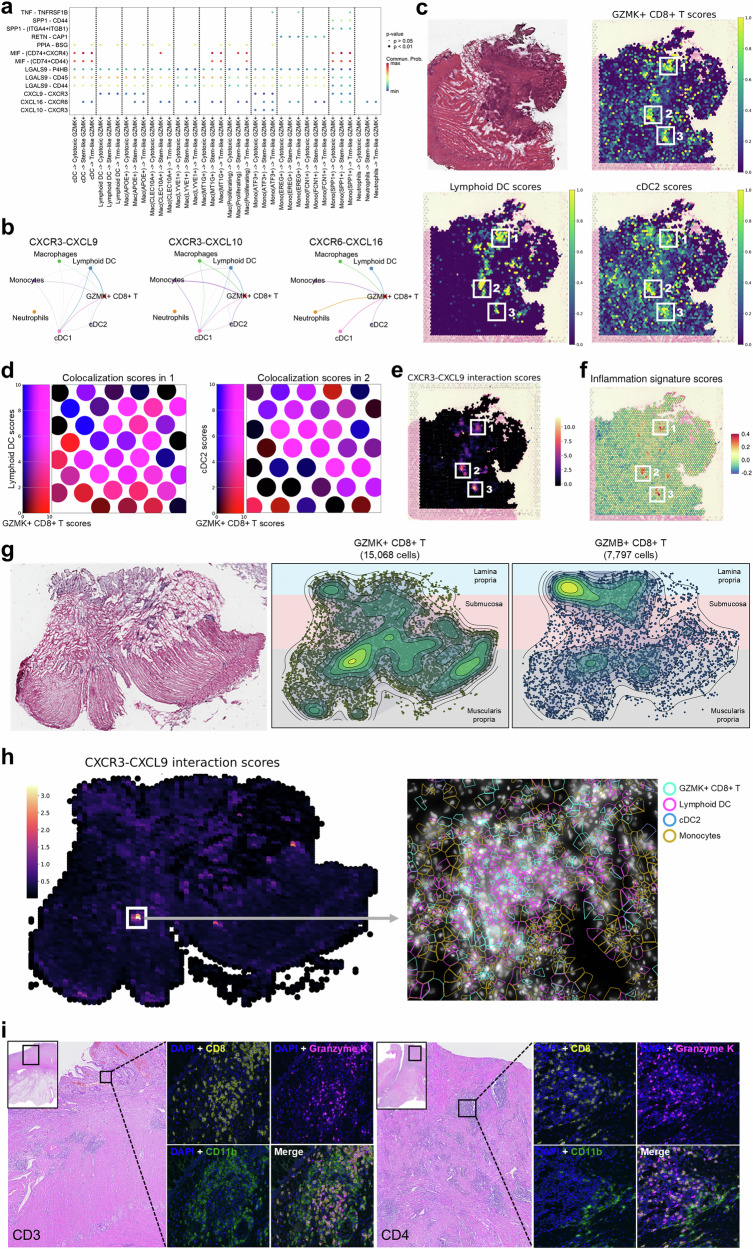
Colocalization and interaction networks between GZMK^+^CD8^+^ T cells and myeloid subsets. **a**, Dot plot illustrating receptor–ligand interactions between GZMK^+^ subclusters (receptors) and myeloid subsets (ligands). Dot size indicates the *P*-value, and color denotes the probability of communication. **b**, Circle network diagram depicting CXCR3–CXCL9/10 and CXCR6–CXCL16 interactions between GZMK^+^CD8^+^ T cells and myeloid subsets in a publicly available scRNA-seq dataset (PMID:37507073) containing both T cells and myeloid cells. Arrows represent directional flow, and edge thickness indicates the sum of weighted paths between cell types. **c**, Hematoxylin and eosin image of an inflamed small intestinal tissue used in 10× Visium analysis and cell-type probabilities assigned to each spot. Three regions with high GZMK^+^CD8^+^ T scores and myeloid subset scores are labeled as 1, 2, and 3. **d**, Spatial feature plots highlighting colocalization of GZMK^+^CD8^+^ T cells with lymphoid dendritic cell (DC) (left) and conventional DC2 (cDC2) (right) in regions 1 and 2 of part **c**. Spots closer to purple indicate regions with higher colocalization between GZMK^+^CD8^+^ T cells and myeloid subsets. **e**, Receptor–ligand interaction scores incorporating spatial coordinates for CXCR3–CXCL9 in the inflamed small intestinal tissue analyzed using 10× Visium. **f**, Signature scores associated with inflammation in the inflamed small intestinal tissue analyzed using 10× Visium. **g**, Hematoxylin and eosin image of an inflamed small intestinal tissue analyzed by 10× Xenium and density plots depicting the spatial distribution patterns of GZMK^+^ and GZMB^+^CD8^+^ T cells. **h**, Receptor–ligand interaction scores for CXCR3–CXCL9 incorporating spatial coordinates (left) in the inflamed small intestinal tissue analyzed by 10× Xenium, and colocalization of GZMK^+^CD8^+^ T cells with myeloid subsets at single-cell resolution in the highlighted region (right). **i**, Representative immunohistochemistry images of inflamed small intestinal tissue from patients with CD stained for CD8, granzyme K (GZMK), and CD11b. DAPI, 4′,6-diamidino-2-phenylindole; GZMB, granzyme B.

To investigate the interactions between GZMK^+^CD8^+^ T cells and other immune cells in the SI microenvironment, we performed a comprehensive analysis of immune cell populations and their spatial relationships. Re-clustering of CD45^+^ immune cells revealed five distinct clusters (Supplementary Fig. 6a). Receptor–ligand interaction analysis highlighted substantial crosstalk between GZMK^+^CD8^+^ T subclusters and myeloid cell populations^25^ (Fig. 4a and Supplementary Fig. 6b,c). Enriched CXCR6–CXCL16 interactions in Trm-like and stem-like GZMK^+^ subclusters (Fig. 4a) suggest a role in tissue retention and maintenance of CD8^+^ T cells. Additionally, we observed notable CXCR3–CXCL9/10 interactions between GZMK^+^ subclusters and lymphoid dendritic cells (DCs), macrophages, and monocytes (Fig. 4a). These findings were corroborated using a publicly available scRNA-seq dataset from patients with CD^27^ (Supplementary Fig. 6d,e), confirming consistent crosstalk between GZMK^+^CD8^+^ T cells and myeloid cells mediated by *CXCR3* and *CXCR6* (Fig. 4b). Importantly, these specific interactions were absent in GZMB^+^CD8^+^ T cells (Supplementary Fig. 6c), indicating a unique activation pathway for GZMK^+^CD8^+^ T cells in local inflammation.

To elucidate the spatial relationships between GZMK^+^CD8^+^ T cells and myeloid cells, we used 10× Visium ST analysis. This approach identified three regions of colocalization between GZMK^+^CD8^+^ T cells and various myeloid subsets (Fig. 4c,d and Supplementary Fig. 7a,b). Within these regions, we observed elevated CXCR3–CXCL9/10 and CXCR6–CXCL16 interactions (Fig. 4e and Supplementary Fig. 7c), as well as high inflammation signatures (Fig. 4f), suggesting enhanced cellular communication in specific tissue niches. The 10× Xenium platform provided single-cell-level resolution ST data, which supported our previous observation of GZMK^+^CD8^+^ T cells being more prevalent than GZMB^+^CD8^+^ T cells in SI tissue and suggested possible spatial distribution patterns (Fig. 4g and Supplementary Fig. 7d). In this sample, GZMK^+^CD8^+^ T cells appeared to be more abundant in the submucosa and muscularis propria regions, hinting at potential differential influences of the SI tissue microenvironment on these CD8^+^ T cell subsets. Receptor–ligand interaction analysis revealed strong CXCR3–CXCL9/10 and CXCR6–CXCL16 interactions within a specific region, accompanied by colocalization of GZMK^+^CD8^+^ T cells with lymphoid DCs, cDC2, and monocytes at the single-cell resolution (Fig. 4h and Supplementary Fig. 7e).

To validate our single-cell and ST findings at the protein level, we performed IHC analysis on inflamed small intestinal tissue samples (*n* = 6) from patients with CD using multiplex staining for CD8, granzyme K, and CD11b. IHC staining confirmed elevated granzyme K protein expression in tissue-infiltrating CD8^+^ T cells within inflammatory regions, consistent with our scRNA-seq observations. Notably, IHC analysis revealed colocalization of granzyme K^+^CD8^+^ T cells with CD11b^+^ myeloid cells within inflammatory foci, providing direct spatial evidence of cell–cell proximity that corroborated our receptor–ligand interaction analyses (Fig. 4i and Supplementary Fig. 7f). The increased density of both granzyme K^+^CD8^+^ T cells and CD11b^+^ myeloid populations in inflammatory regions suggested their coordinated involvement in local inflammatory responses. These protein-level validations across multiple samples corroborated the spatial and transcriptomic evidence of GZMK^+^CD8^+^ T cell infiltration and interaction with myeloid populations in inflamed CD tissues. These findings collectively suggested that GZMK^+^CD8^+^ T cells established close spatial associations and engage in multiple interaction networks with myeloid cells within inflamed SI tissues of patients with CD.

To further refine the spatial characterization of the GZMK^+^ compartment, we next examined the spatial niche of the GZMK^+^GZMB^+^ double-positive subset. Using 10× Xenium ST, we found that GZMK^+^GZMB^+^ double-positive cells localized predominantly to inflammatory foci within the small intestinal submucosa and muscularis propria, exhibiting spatial distribution patterns highly similar to those of GZMK-only cells (Supplementary Fig. 7g). Consistent with this, distance-based spatial analyses using single-cell coordinates indicated that distances from GZMK^+^GZMB^+^ cells to GZMK-only cells were closer than those to GZMB^+^CD8^+^ T cells (Supplementary Fig. 7h). Similar to GZMK-only cells, GZMK^+^GZMB^+^ cells colocalized with myeloid populations, including monocytes and DCs, within regions enriched for inflammatory gene signatures. Notably, they were also enriched in areas with elevated CXCL9/10 and CXCL16 expression, mirroring the chemokine-associated spatial niches observed for GZMK-only cells and consistent with chemokine-mediated retention. These findings indicated that GZMK^+^GZMB^+^ cells occupy inflammatory niches shared with GZMK^+^ populations within the intestinal microenvironment.

### Amelioration of intestinal inflammation by GZMK blockade

**Fig. 5:**
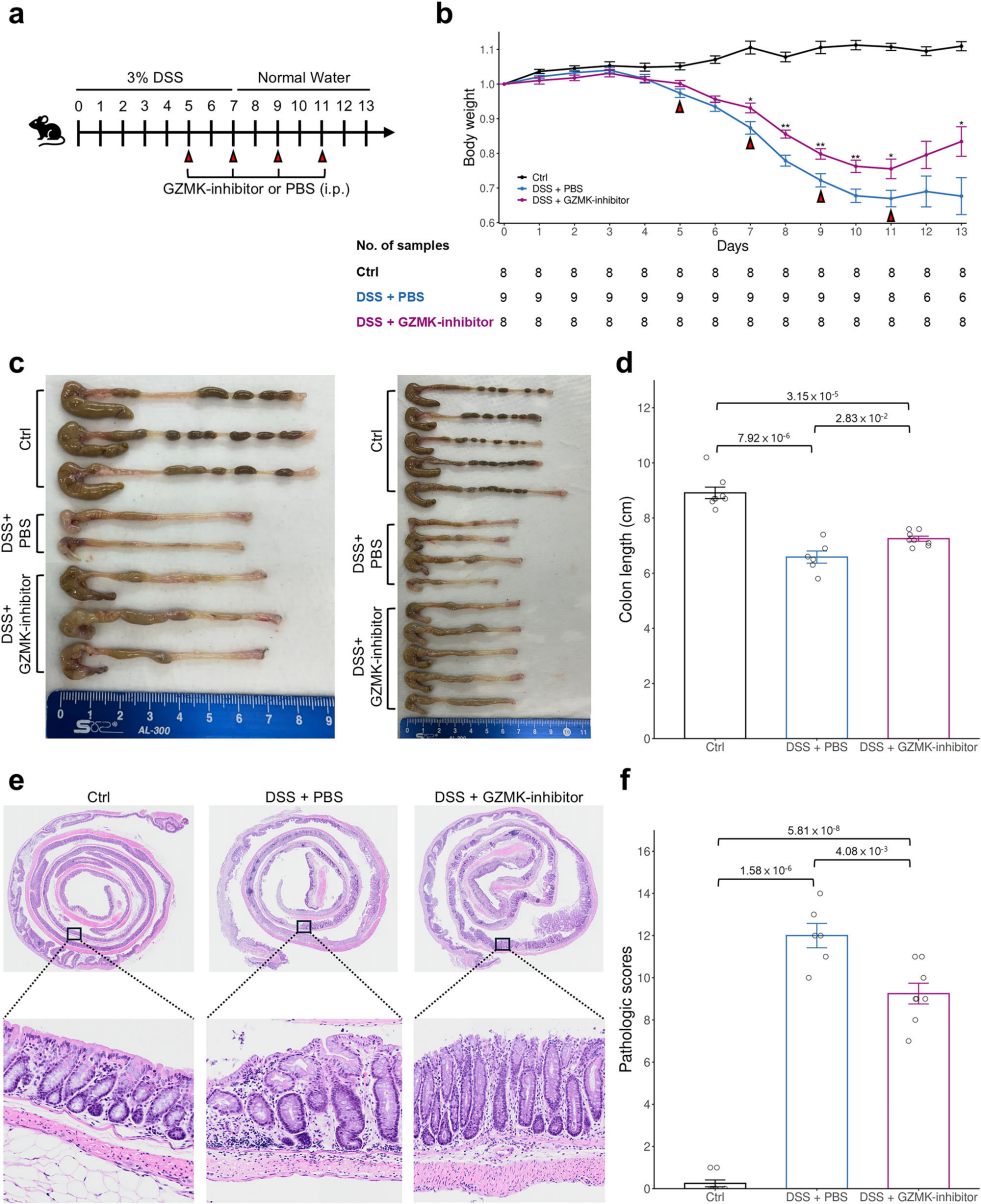
Therapeutic effects of GZMK inhibition in a DSS-induced colitis model. **a**, Experimental timeline for the dextran sulfate sodium (DSS)-induced colitis model. The control (Ctrl) group received only normal drinking water throughout the experiment. By contrast, the DSS + PBS and DSS + GZMK inhibitor groups were administered 3% DSS in drinking water for 7 days, followed by normal water. During the recovery phase, mice were intraperitoneally injected with either GZMK inhibitor or PBS every other day. **b**, Daily body weight change during the experiment (mean ± standard error of the mean; *n* = 6–9 per group). The table below the graph shows the number of surviving mice at each time point in each group. **c**, Images of colons collected from all mice in each group at day 13. Shown are replicate results from two independent experiments, with experiment 1 on the left and experiment 2 on the right. **d**, Quantification of colon length (mean ± standard error of the mean; *n* = 6–8 per group). **e**, Representative hematoxylin and eosin-stained colon sections showing histological changes in each group. Lower panels show magnified views of the boxed regions above. **f**, Pathological scores based on histological assessment (mean ± standard error of the mean; *n* = 6–8 per group). Statistical analysis was performed using unpaired two-tailed Student’s *t* test. GZMK, granzyme K; i.p., intraperitoneal.

To investigate the functional contribution of GZMK to intestinal injury, a DSS-induced colitis model was used, in which mice were intraperitoneally administered either a GZMK inhibitor (that is, PPACK) or PBS every 2 days (Fig. 5a). Mice treated with the GZMK inhibitor exhibited significantly attenuated loss of body weight compared with the PBS-treated group (Fig. 5b), suggesting that GZMK blockade may directly modulate intestinal inflammation. Moreover, the colon shortening typically observed following DSS administration was partially restored in the GZMK-inhibitor-treated group (Fig. 5c,d). Histological analysis further revealed reduced tissue damage and better preservation of mucosal architecture in the GZMK-inhibitor-treated group compared with mice treated with the PBS (Fig. 5e). Consistently, pathological scores were significantly lower in the GZMK-inhibitor-treated mice (Fig. 5f and Supplementary Table 5), indicating attenuation of tissue injury. During the experimental period, three mice in the PBS-treated group reached end points and were excluded from the final statistical analysis owing to severe colitis-related mortality. Despite this increased disease severity in the PBS-treated group, GZMK-inhibitor treatment demonstrated significant therapeutic efficacy, suggesting a substantial protective effect against DSS-induced intestinal inflammation.

## Discussion

In this study, we used an integrated approach combining scRNA-seq, scTCR-seq, and ST to investigate heterogeneity of CD8^+^ T cells and tissue distribution in patients with CD. Our findings revealed distinct transcriptional and functional states of GZMK^+^CD8^+^ T cells, highlighting their involvement in intestinal inflammation.

Our observations of GZMK^+^CD8^+^ T cells in the SI aligned with CD103^−^ Trm populations found to persist in tissues for years in transplant recipients^47^. GZMK^+^CD8^+^ T cells shared numerous features with CD103^−^ Trm populations, including elevated *GZMK* and *CXCR6* expression, enhanced cytotoxicity compared with CD103^+^ Trm cells, and CXCR3-mediated recruitment to inflamed areas^6,32,48^. Given the role of CD103^−^ Trm cells as primary responders to secondary infections in the intestine^49^, we postulated that GZMK^+^CD8^+^ T cells might serve a similar function in inflammatory conditions. Furthermore, in the context of chronic recurrent inflammation, analysis of nasal polyp samples from initial and recurrent surgeries in patients with chronic rhinosinusitis, conducted at least 7 months apart, revealed persistent GZMK^+^CD8^+^ T cell clonotypes in the tissue^7^. This suggested their crucial role in perpetuating and driving chronic inflammation. Collectively, these findings indicated that local tissue residency might occur through diverse mechanisms, not solely dependent on CD103 expression^50^, and suggested that GZMK^+^CD8^+^ T cells could potentially represent a subset of T cells with tissue-resident features contributing to chronic inflammation in CD. Beyond their role in local cellular interactions and maintenance of tissue-resident populations, CXCR6 and CXCR3 also facilitate the initial recruitment of circulating T cells to inflamed intestinal tissues. The dual functions of these chemokine receptors in both trafficking and retention highlight their multifaceted contributions to CD pathogenesis and suggest that therapeutic targeting of these pathways could address multiple stages of T cell accumulation in affected tissues.

The observed high tissue residency and low circulating capacity of GZMK^+^CD8^+^ T cells in the SI of CD suggested that these cells might adapt to and persist in the inflamed environment. These results highlighted the challenges in managing T cells in inflamed tissue, as SI GZMK^+^CD8^+^ T cells could remain even when therapies inhibit new T cell recruitment to the intestine. Considering the propensity of GZMK^+^CD8^+^ T cells to persist in tissues, we investigated their role in the local inflammatory environment. Our analysis of inflamed intestinal tissues revealed significant CXCR3–CXCL9/10 interactions between GZMK^+^CD8^+^ T cells and myeloid cells. These findings suggested that GZMK^+^CD8^+^ T cells might be a key target for CXCR3 blockade, potentially explaining its efficacy in reducing T cell infiltration and tissue damage observed in recent studies^49,51^. Thus, their molecular profiles promoted tissue retention and myeloid cell interactions, suggesting GZMK^+^CD8^+^ T cells as a promising target for CD treatment.

This study had several limitations. Focussing on SI tissues and peripheral blood of patients with CD restricted comparisons to healthy conditions. Our analysis of only CD45^+^ immune cells left interactions with non-immune populations, particularly fibroblasts, unexplored. Our single-cell approach provided only a static snapshot, and we observed high clonal relationships between GZMK^+^CD8^+^ T cells and CD8^+^ Trm cells without direct evidence of differentiation. The reduced cytotoxic signatures of GZMK⁺CD8⁺ T cells inferred from transcriptomic profiling were not corroborated by functional killing assays, and redirected cytotoxicity experiments with isolated intestinal T cells would be required for definitive validation. In addition, PPACK acted as a broad‑spectrum serine protease inhibitor with potential off‑target effects, and the DSS‑induced colitis model — driven largely by epithelial injury and innate immunity and predominantly affecting the colon — did not fully recapitulate the T cell‑mediated, small‑intestinal pathology typical of CD. Taken together with the tissue specificity of immune responses reported in non‑intestinal GZMK‑deficient or inhibitor models^7,8^, these considerations indicated that validation of GZMK’s role in CD would require T cell‑dependent models and complementary genetic approaches.

On the basis of these limitations, several research priorities emerged from this study. First, disease models centered on adaptive immunity, such as the CD45RB^high^ adoptive transfer model, would provide a complementary platform to evaluate whether GZMK⁺CD8⁺ T cells contribute to the exacerbation of intestinal inflammation in a T cell-dependent context. Second, GZMK‑specific genetic perturbation — such as knockout or conditional deletion models — would be expected to provide more precise mechanistic insights than pharmacological inhibition and to clarify GZMK‑dependent pathways in tissue damage and chronic inflammation. Third, integrative single‑cell and spatial approaches that explicitly include stromal and epithelial compartments could define how GZMK⁺CD8⁺ T cells communicate with intestinal fibroblasts and other non‑immune cells to promote tissue remodeling and fibrosis in CD. Finally, preclinical studies targeting the CXCR3/CXCR6 axes or GZMK enzymatic activity in these T cell-dependent models would be essential to determine whether selective modulation of GZMK⁺CD8⁺ T cells can safely limit tissue‑resident accumulation and ameliorate CD, thereby establishing the translational potential of the present findings.

In conclusion, this study identifies a distinct GZMK^+^CD8^+^ T population in patients with CD that exhibits unique tissue residency features and may contribute to local inflammation. These cells, sharing select features with CD103^−^ Trm populations, exemplify the diverse nature of disease-associated memory CD8^+^ T cells involved in chronic inflammatory processes. Their specific transcriptional profile and chemokine receptor expression patterns may promote retention in inflamed tissues and interactions with myeloid cells, potentially contributing to ongoing inflammation. Our findings provide new insights into CD8^+^ T cell heterogeneity in CD and highlight these T cell subsets as potential targets for further investigation in the context of chronic inflammation in CD.

## Supplementary information

Supplementary Information

## Data Availability

All sequencing datasets generated in this study have been deposited in the NCBI database under the accession number GSE289496 for both the scRNA-seq and spatial transcriptomic data. All other data generated or analyzed in this study, aside from the scRNA-seq and spatial transcriptomic data, are available in the article and its online supplementary material.
