# Supplementary material for: Granzyme K CD8⁺ T cells with tissue-resident features promote intestinal inflammation in patients with Crohn’s disease
Source: Exp Mol Med. 2026 Jul 6;58(7):2280–92. doi: 10.1038/s12276-026-01763-7 (PMC13434261; doi:10.1038/s12276-026-01763-7)
Supplement: Supplementary file 1 [file 12276_2026_1763_MOESM1_ESM.pdf]

SUPPLEMENTARY MATERIAL

Granzyme K CD8^{+} T Cells with Tissue-Resident Features Promote Intestinal Inflammation in Patients with Crohn’s Disease

Supplementary Figures 1−7 and Supplementary Tables 1-5

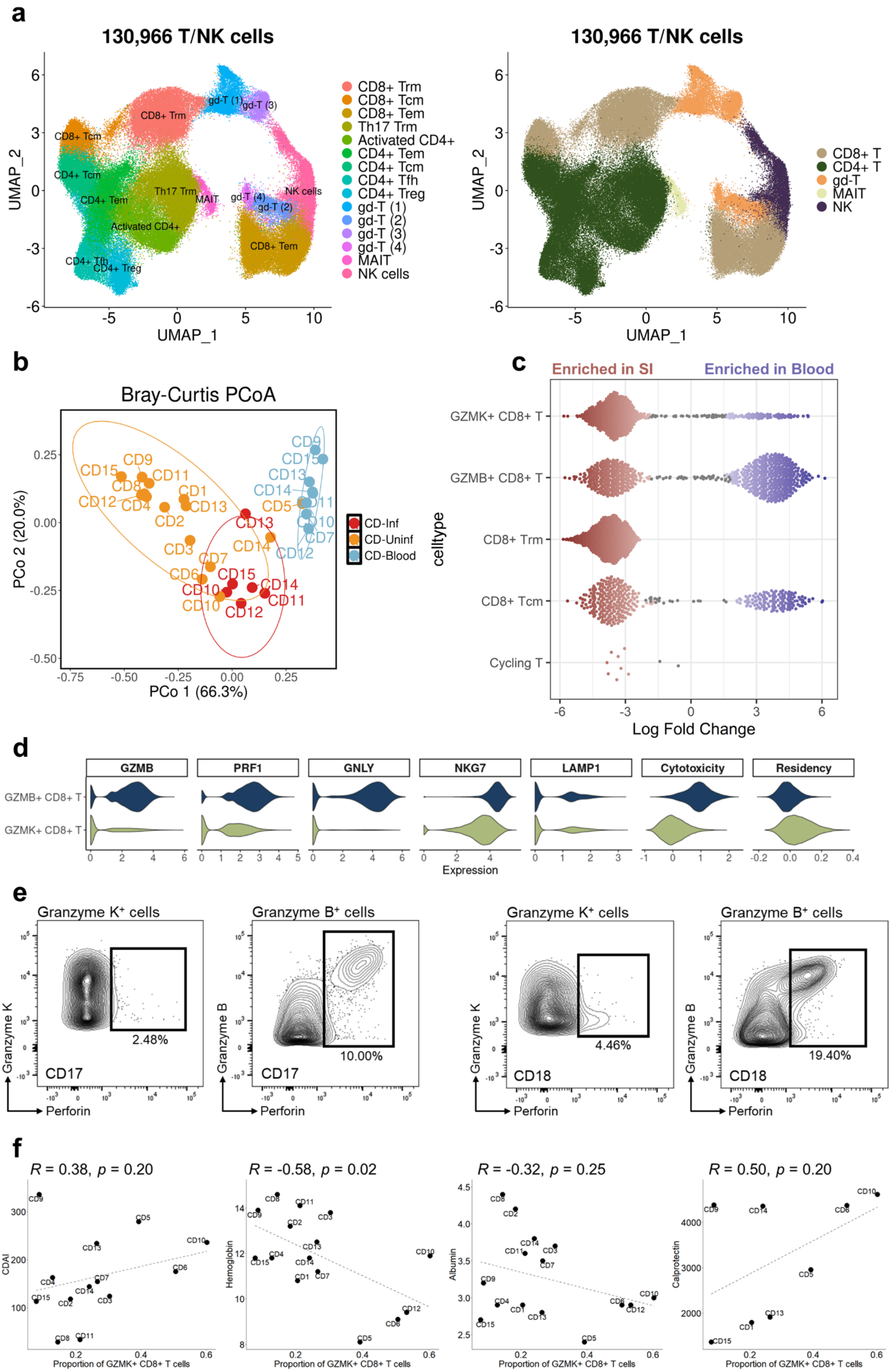

a
130,966 T/NK cells
UMAP_1
UMAP_2
CD8+ Trm
CD8+ Tcm
CD8+ Tem
Th17 Trm
Activated CD4+
CD4+ Tem
CD4+ Tcm
CD4+ Tfh
CD4+ Treg
gd-T (1)
gd-T (2)
gd-T (3)
gd-T (4)
MAIT
NK cells
130,966 T/NK cells
CD8+ T
CD4+ T
gd-T
MAIT
NK
b
Bray-Curtis PCoA
PCo 1 (66.3%)
PCo 2 (20.0%)
CD-Inf
CD-Uninf
CD-Blood
c
Enriched in SI
Enriched in Blood
GZMK+ CD8+ T
GZMB+ CD8+ T
CD8+ Trm
CD8+ Tcm
Cycling T
celltype
Log Fold Change
d
GZMB
PRF1
GNLY
NKG7
LAMP1
Cytotoxicity
Residency
GZMB+ CD8+ T
GZMK+ CD8+ T
Expression
e
Granzyme K+ cells
Granzyme B+ cells
Granzyme K+ cells
Granzyme B+ cells
2.48%
10.00%
4.46%
19.40%
CD17
CD17
CD18
CD18
Granzyme K
Granzyme B
Perforin
f
R = 0.38, p = 0.20
R = -0.58, p = 0.02
R = -0.32, p = 0.25
R = 0.50, p = 0.20
CDAI
Hemoglobin
Albumin
Calprotectin
Proportion of GZMK+ CD8+ T cells

Supplementary Fig. 1. a Uniform manifold approximation and projection (UMAP) plot of the entire T/NK cell compartment (left) and UMAP projection colored by 5 major T/NK subsets (right), including $CD8^+$ T cells (CD8+ T), $CD4^+$ T cells (CD4+ T), gamma-delta T cells (gd-T), mucosal-associated invariant T cells (MAIT), and NK cells (NK). **b** Principal coordinate analysis (PCoA) showing sample-level differences in the proportion of $CD8^+$ T cells across groups. **c** Beeswarm plot depicting tissue-specific differential abundance for each cell type, based on Milo algorithm. **d** Violin plots showing the expression levels of cytotoxic effector genes (*GZMB*, *PRF1*, *GNLY*, *NKG7*), the degranulation marker *LAMP1*, and module scores for cytotoxicity and tissue residency in $GZMK^+$ and $GZMB^+CD8^+$ T cells. **e** Flow cytometry plots showing perforin protein expression in granzyme K^+ and granzyme B^+ cells from small intestinal tissues of two patients with Crohn's disease (CD). **f** Associations between the proportion of $GZMK^+CD8^+$ T cells and clinical disease severity parameters, including Clinical Disease Activity Index (CDAI), hemoglobin, albumin, and fecal calprotectin, in patients with CD.

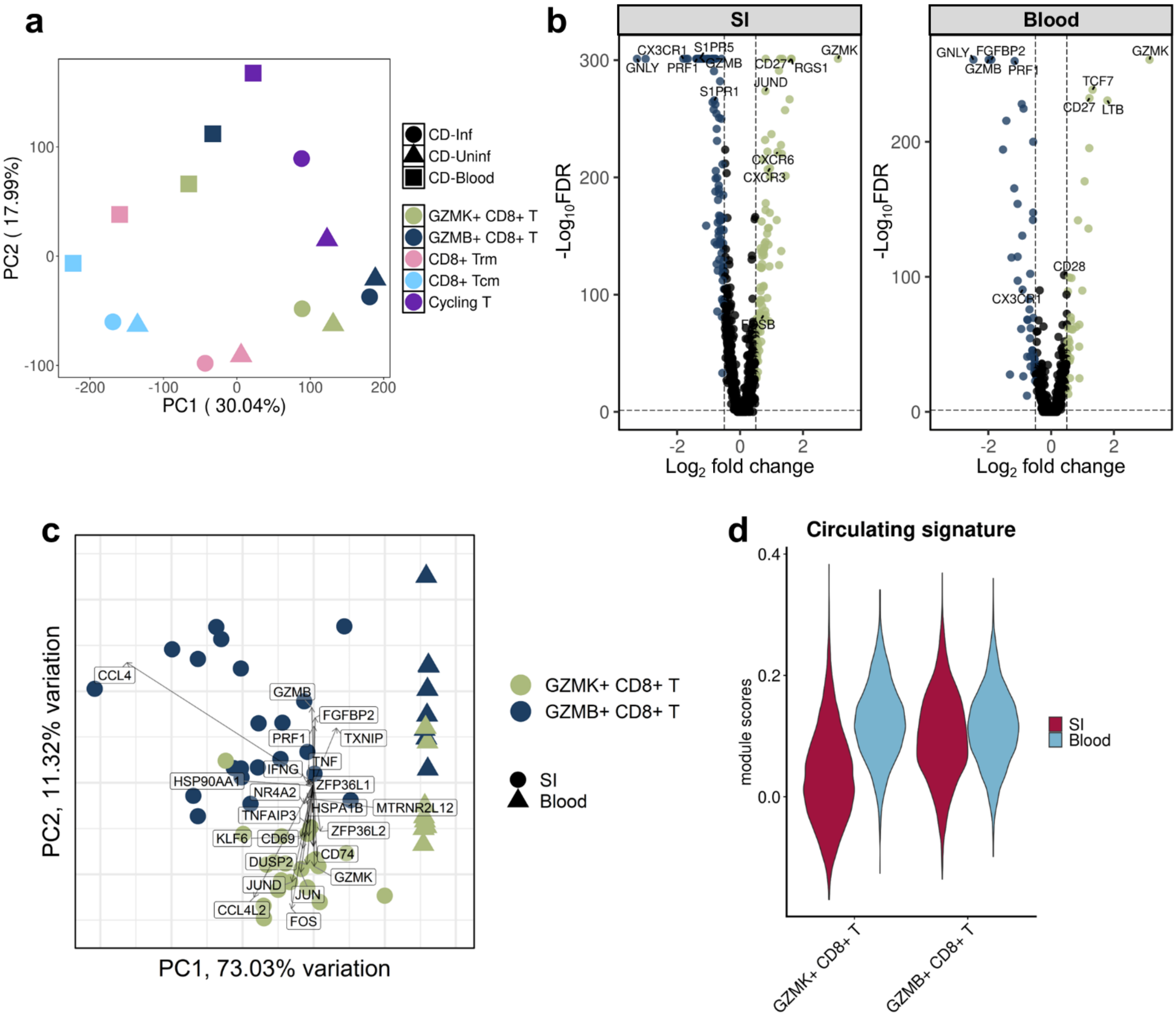

Supplementary Fig. 2. a Principal component analysis (PCA) plot illustrating expression profiles of CD8$^+$ T cell clusters across tissues. **b** Volcano plots displaying differential expression analysis results between GZMK$^+$ and GZMB$^+$CD8$^+$ T cells in small intestine (SI) and blood. **c** PCA plot highlighting key markers associated with differences between GZMK$^+$ and GZMB$^+$CD8$^+$ T cells. **d** Circulating signature scores of GZMK$^+$ and GZMB$^+$CD8$^+$ T cells in SI and blood.

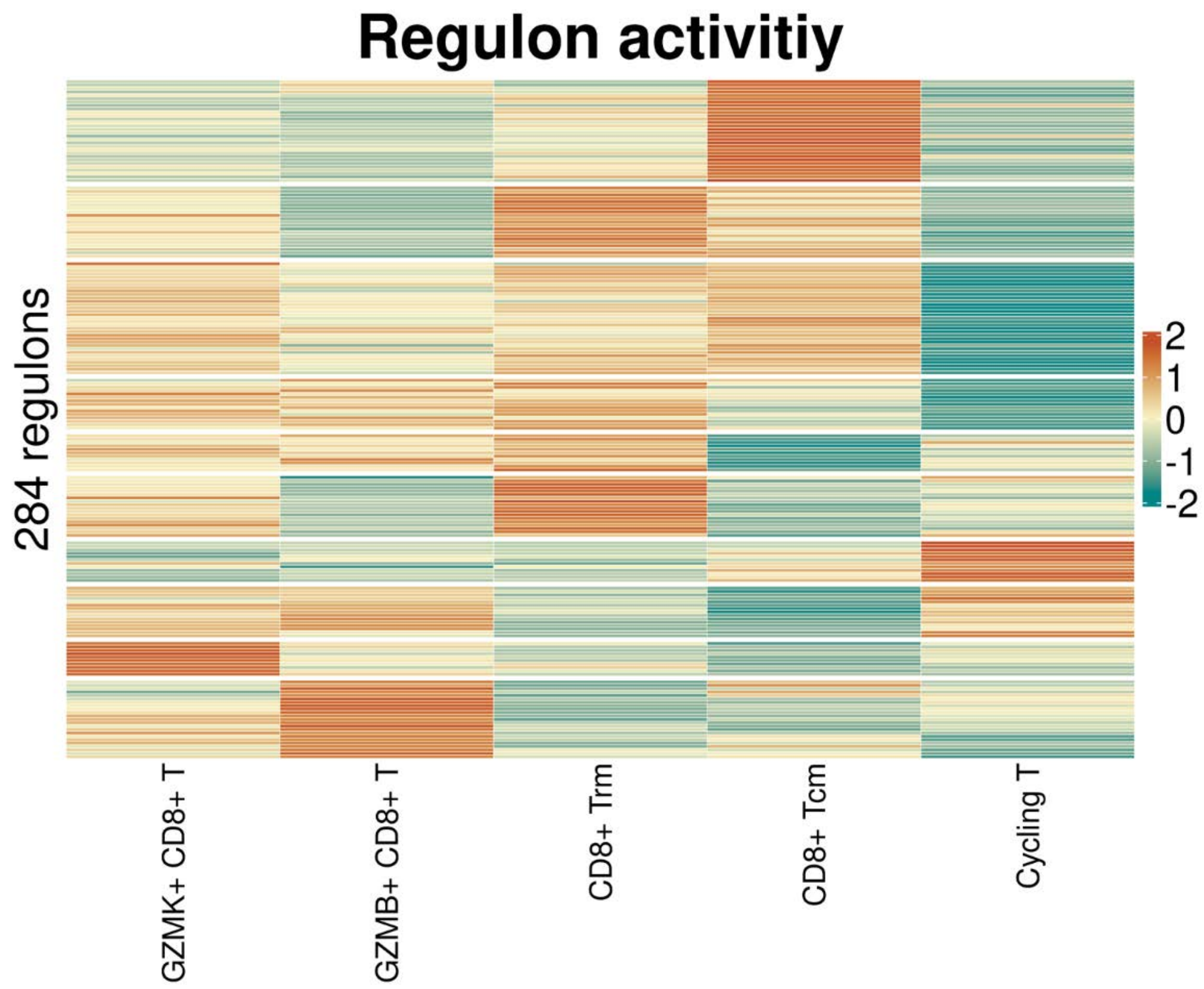

Supplementary Fig. 3. Heatmap visualizing relative regulon activity for all detected transcription factors (TF) modules in CD8$^+$ T cell clusters.

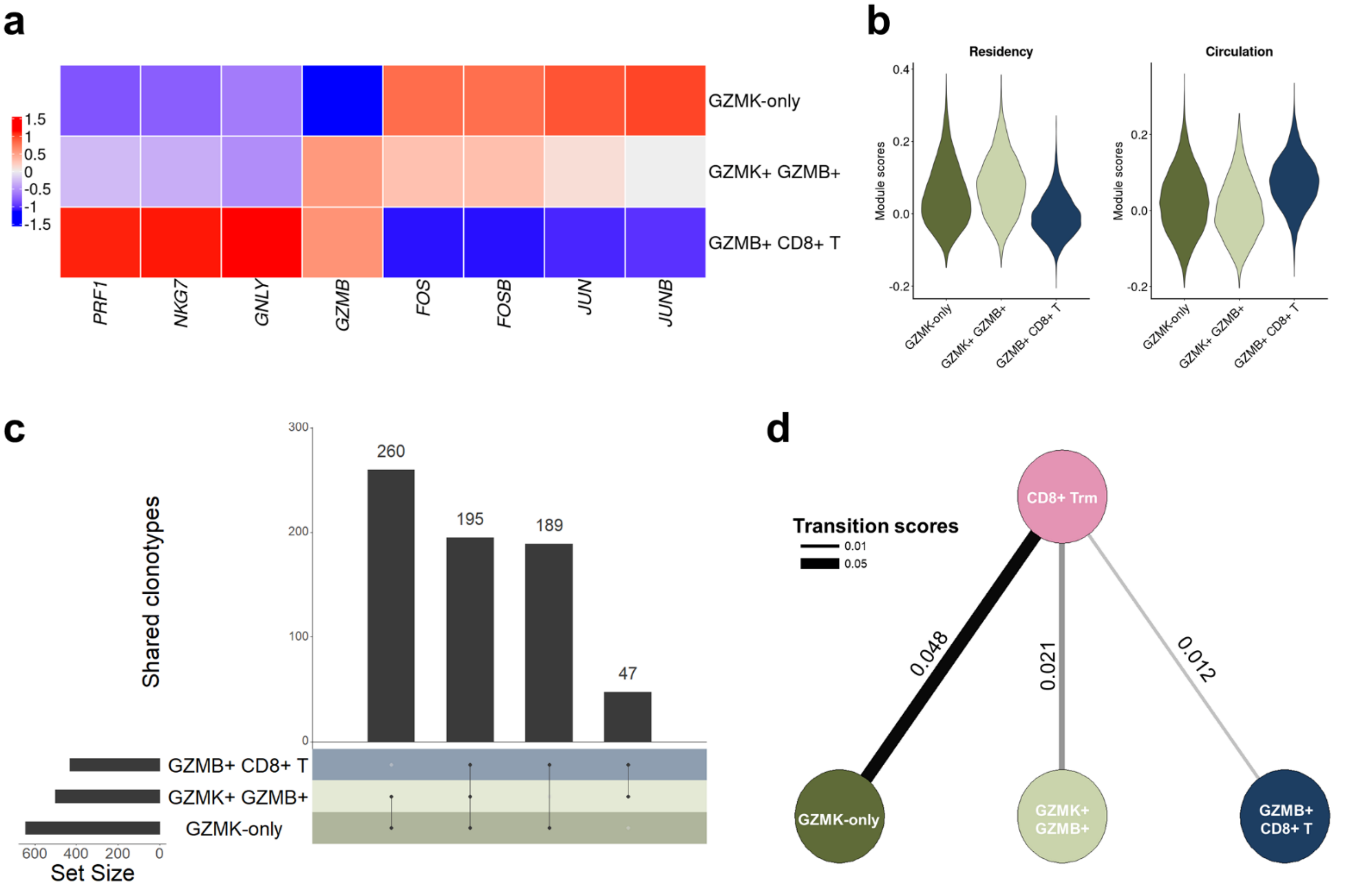

Supplementary Fig. 4. a A heatmap showing the expression levels of selected marker genes for GZMK-only, $GZMK^+GZMB^+$, and $GZMB^+CD8^+$ T cell subsets. **b** Violin plots showing tissue residency and circulation module scores of GZMK-only, $GZMK^+GZMB^+$, and $GZMB^+CD8^+$ T cells. **c** UpSet plots representing the number of shared clonotypes between cells of GZMK-only, $GZMK^+GZMB^+$, and $GZMB^+CD8^+$ T cell clusters. Black circles connected by a black line indicate the presence of shared clonotypes among the connected clusters. The number above each top bar denotes the number of shared clonotypes between the corresponding clusters. The bar on the left of each cluster represents the number of clonotypes shared with at least one other cluster. **d** Cross-cluster phenotypic transition scores between GZMK-only, $GZMK^+GZMB^+$, $GZMB^+CD8^+$ T cells, and $CD8^+$ Trm cells. The thickness of each connecting line represents the degree of TCR clonotype sharing between two different clusters.

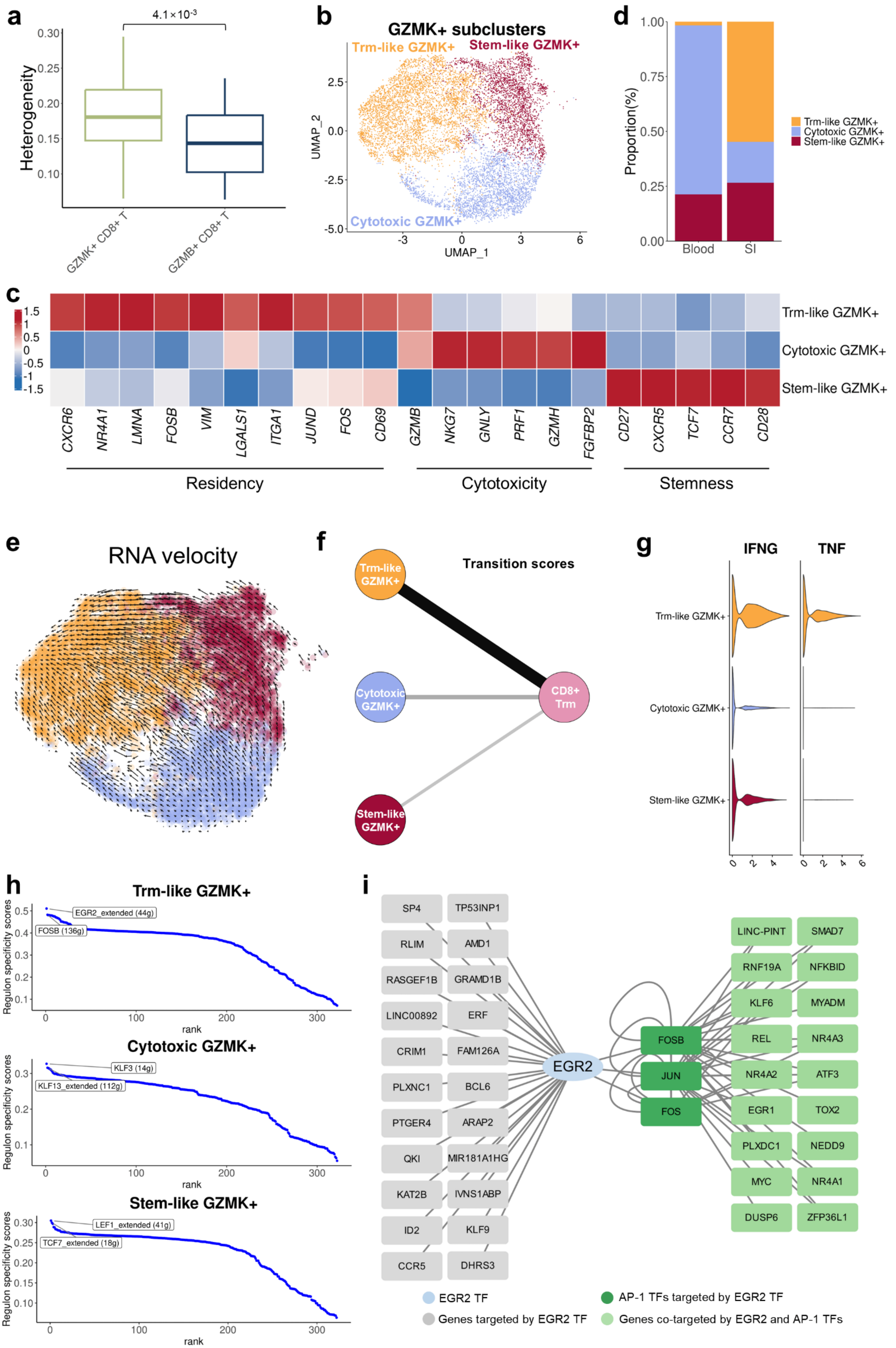

a
4.1×10-3
Heterogeneity
GZMK+ CD8+ T
GZMB+ CD8+ T
b
GZMK+ subclusters
Trm-like GZMK+
Stem-like GZMK+
Cytotoxic GZMK+
UMAP_1
UMAP_2
d
Proportion(%)
Blood
SI
Trm-like GZMK+
Cytotoxic GZMK+
Stem-like GZMK+
c
CXCR6
NR4A1
LMNA
FOSB
VIM
LGALS1
ITGA1
JUND
FOS
CD69
GZMB
NKG7
GNLY
PRF1
GZMH
FGFBP2
CD27
CXCR5
TCF7
CCR7
CD28
Residency
Cytotoxicity
Stemness
e
RNA velocity
f
Transition scores
Trm-like GZMK+
Cytotoxic GZMK+
Stem-like GZMK+
CD8+ Trm
g
IFNG
TNF
h
Trm-like GZMK+
EGR2_extended (44g)
FOSB (136g)
Cytotoxic GZMK+
KLF3 (14g)
KLF13_extended (112g)
Stem-like GZMK+
LEF1_extended (41g)
TCF7_extended (18g)
Regulon specificity scores
rank
i
SP4
TP53INP1
RLIM
AMD1
RASGEF1B
GRAMD1B
LINC00892
ERF
CRIM1
FAM126A
PLXNC1
BCL6
PTGER4
ARAP2
QKI
MIR181A1HG
KAT2B
IVNS1ABP
ID2
KLF9
CCR5
DHRS3
EGR2
FOSB
JUN
FOS
LINC-PINT
SMAD7
RNF19A
NFKBID
KLF6
MYADM
REL
NR4A3
NR4A2
ATF3
EGR1
TOX2
PLXDC1
NEDD9
MYC
NR4A1
DUSP6
ZFP36L1
EGR2 TF
Genes targeted by EGR2 TF
AP-1 TFs targeted by EGR2 TF
Genes co-targeted by EGR2 and AP-1 TFs

Supplementary Fig. 5. a Comparison of heterogeneity between GZMK$^+$ and GZMB$^+$CD8$^+$ T subsets across individual samples. **b** UMAP plot of GZMK$^+$ subclusters. **c** A heatmap showing the expression levels of selected marker genes for GZMK$^+$ subclusters. **d** Proportions of the three GZMK$^+$ subclusters in blood and SI. **e** UMAP plot of RNA velocities illustrating the transcriptional dynamics and trajectory of GZMK$^+$ subclusters. **f** Cross-cluster phenotypic transition scores between GZMK$^+$ subclusters and CD8$^+$ Trm. The thickness of the line represents a measure of clonotype sharing between two different clusters. **g** Violin plots showing expression levels of *IFNG* and *TNF* in GZMK$^+$ subclusters. **h** Ranking of regulons within each GZMK$^+$ subcluster based on regulon specificity scores. **i** Network visualization of EGR2 and its predicted target genes from the gene regulatory network analysis. Genes highlighted in light green are predicted target genes of both EGR2 and AP-1 TFs.

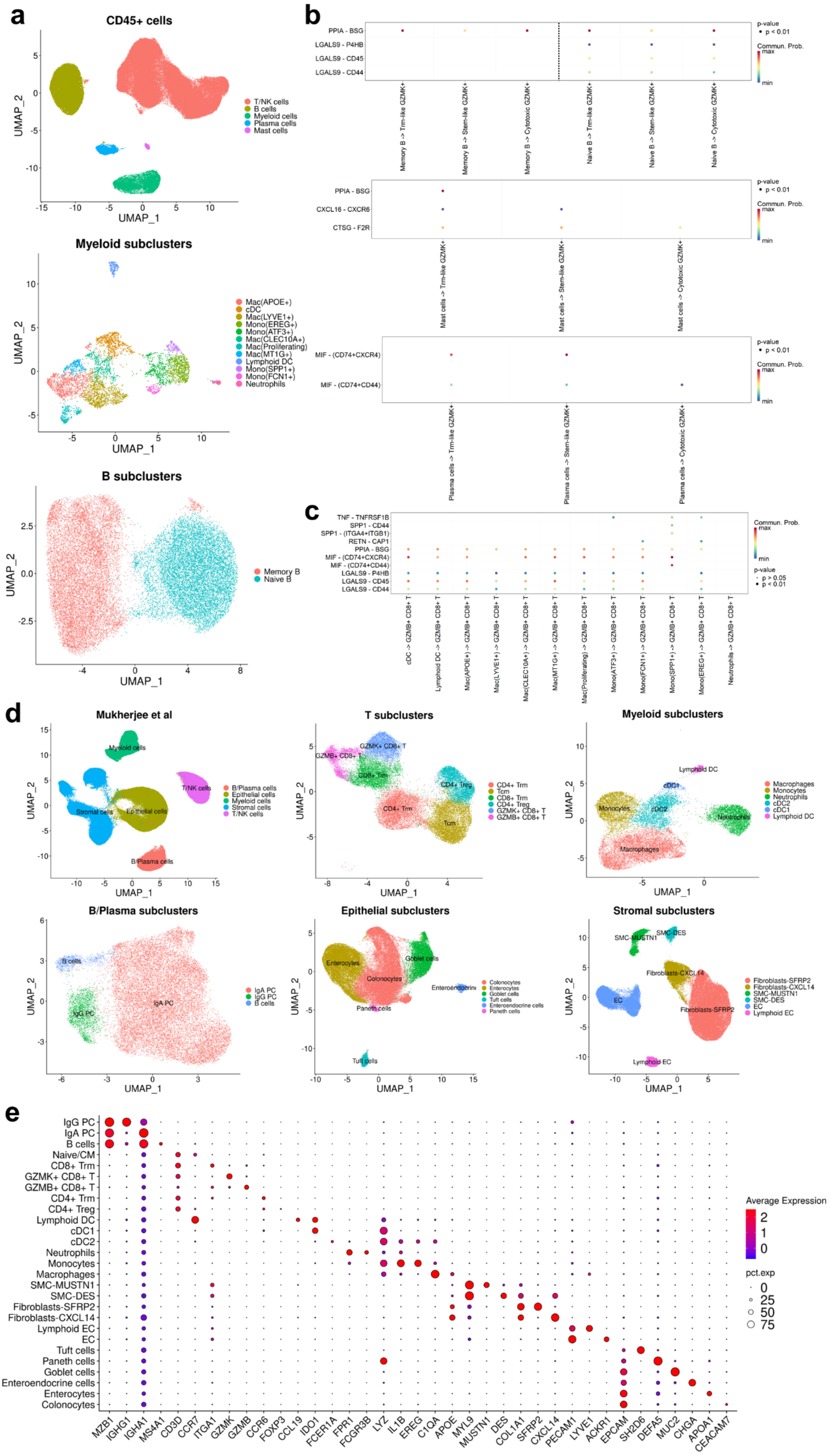

a
CD45+ cells
UMAP_1
UMAP_2
T/NK cells
B cells
Myeloid cells
Plasma cells
Mast cells
Myeloid subclusters
Mac(APOE+)
cDC
Mac(LYVE1+)
Mono(EREG+)
Mono(ATF3+)
Mac(CLEC10A+)
Mac(Proliferating)
Mac(MT1G+)
Lymphoid DC
Mono(SPP1+)
Mono(FCN1+)
Neutrophils
B subclusters
Memory B
Naive B
b
PPIA - BSG
LGALS9 - P4HB
LGALS9 - CD45
LGALS9 - CD44
p-value
p < 0.01
Commun. Prob.
max
min
Memory B -> Trm-like GZMK+
Memory B -> Stem-like GZMK+
Memory B -> Cytotoxic GZMK+
Naive B -> Trm-like GZMK+
Naive B -> Stem-like GZMK+
Naive B -> Cytotoxic GZMK+
CXCL16 - CXCR6
CTSG - F2R
Mast cells -> Trm-like GZMK+
Mast cells -> Stem-like GZMK+
Mast cells -> Cytotoxic GZMK+
MIF - (CD74+CXCR4)
MIF - (CD74+CD44)
Plasma cells -> Trm-like GZMK+
Plasma cells -> Stem-like GZMK+
Plasma cells -> Cytotoxic GZMK+
c
TNF - TNFRSF1B
SPP1 - CD44
SPP1 - (ITGA4+ITGB1)
RETN - CAP1
PPIA - BSG
MIF - (CD74+CXCR4)
MIF - (CD74+CD44)
LGALS9 - P4HB
LGALS9 - CD45
LGALS9 - CD44
p > 0.05
p < 0.01
cDC -> GZMB+ CD8+ T
Lymphoid DC -> GZMB+ CD8+ T
Mac(APOE+) -> GZMB+ CD8+ T
Mac(LYVE1+) -> GZMB+ CD8+ T
Mac(CLEC10A+) -> GZMB+ CD8+ T
Mac(MT1G+) -> GZMB+ CD8+ T
Mac(Proliferating) -> GZMB+ CD8+ T
Mono(ATF3+) -> GZMB+ CD8+ T
Mono(FCN1+) -> GZMB+ CD8+ T
Mono(SPP1+) -> GZMB+ CD8+ T
Mono(EREG+) -> GZMB+ CD8+ T
Neutrophils -> GZMB+ CD8+ T
d
Mukherjee et al
B/Plasma cells
Epithelial cells
Myeloid cells
Stromal cells
T/NK cells
T subclusters
CD4+ Trm
Tcm
CD8+ Trm
CD4+ Treg
GZMK+ CD8+ T
GZMB+ CD8+ T
Myeloid subclusters
Macrophages
Monocytes
Neutrophils
cDC2
cDC1
Lymphoid DC
B/Plasma subclusters
IgA PC
IgG PC
B cells
Epithelial subclusters
Colonocytes
Enterocytes
Goblet cells
Tuft cells
Enteroendocrine cells
Paneth cells
Stromal subclusters
Fibroblasts-SFRP2
Fibroblasts-CXCL14
SMC-MUSTN1
SMC-DES
EC
Lymphoid EC
e
IgG PC
IgA PC
B cells
Naive/CM
CD8+ Trm
GZMK+ CD8+ T
GZMB+ CD8+ T
CD4+ Trm
CD4+ Treg
Lymphoid DC
cDC1
cDC2
Neutrophils
Monocytes
Macrophages
SMC-MUSTN1
SMC-DES
Fibroblasts-SFRP2
Fibroblasts-CXCL14
Lymphoid EC
EC
Tuft cells
Paneth cells
Goblet cells
Enteroendocrine cells
Enterocytes
Colonocytes
MZB1
IGHG1
IGHA1
MS4A1
CD3D
CCR7
ITGA1
GZMK
GZMB
CCR6
FOXP3
CCL19
IDO1
FCER1A
FPR1
FCGR3B
LYZ
IL1B
EREG
C1QA
APOE
MYL9
MUSTN1
DES
COL1A1
SFRP2
CXCL14
PECAM1
LYVE1
ACKR1
EPCAM
SH2D6
DEFA5
MUC2
CHGA
APOA1
CEACAM7
Average Expression
pct.exp

Supplementary Fig. 6. a UMAP plots of the CD45$^+$ cell compartment and subclusters of the myeloid cell and B cell clusters within the CD45$^+$ clusters. Plasma cell and mast cell clusters were not subclustered due to the limited number of cells. **b** Dot plot showing receptor-ligand interactions between GZMK$^+$ subclusters and other immune cells based on the CellChat database, with dot size representing p-values and color indicating communication probability. **c** Dot plot depicting receptor-ligand interactions between GZMB$^+$CD8$^+$ T cell cluster and myeloid subsets using the CellChat database. **d** UMAP plot of a public scRNA-seq dataset used to validate interactions between GZMK$^+$CD8$^+$ T cells and other immune cells. **e** Dot plot illustrating the expression profiles of marker genes for each cell type in the public scRNA-seq dataset.

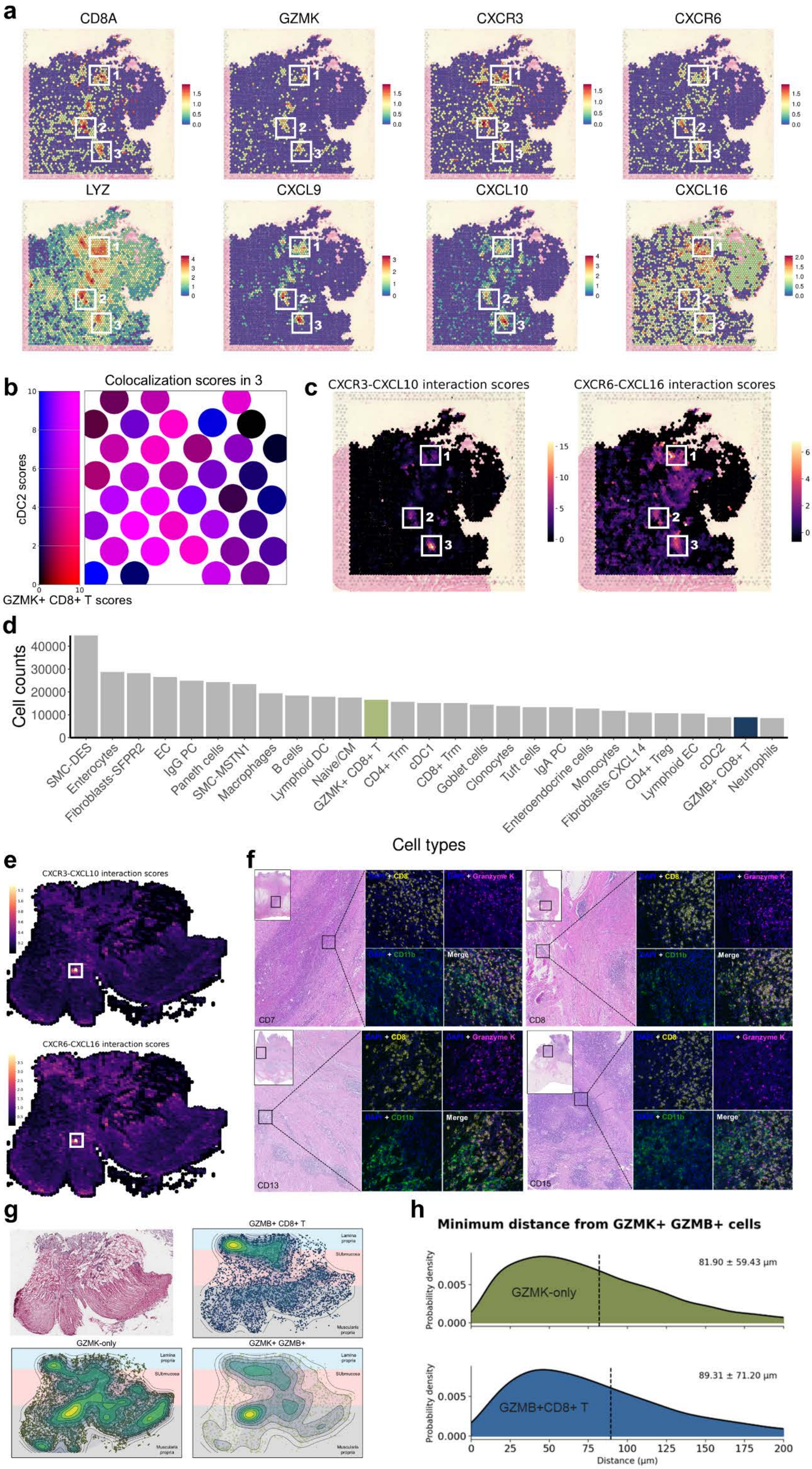
a
CD8A
GZMK
CXCR3
CXCR6
LYZ
CXCL9
CXCL10
CXCL16
b
Colocalization scores in 3
cDC2 scores
GZMK+ CD8+ T scores
c
CXCR3-CXCL10 interaction scores
CXCR6-CXCL16 interaction scores
d
Cell counts
SMC-DES
Enterocytes
Fibroblasts-SFPR2
EC
IgG PC
Paneth cells
SMC-MSTN1
Macrophages
B cells
Lymphoid DC
Naive/CM
GZMK+ CD8+ T
CD4+ Trm
cDC1
CD8+ Trm
Goblet cells
Clonocytes
Tuft cells
IgA PC
Enteroendocrine cells
Monocytes
Fibroblasts-CXCL14
CD4+ Treg
Lymphoid EC
cDC2
GZMB+ CD8+ T
Neutrophils
Cell types
e
CXCR3-CXCL10 interaction scores
CXCR6-CXCL16 interaction scores
f
DAPI + CD8
DAPI + Granzyme K
DAPI + CD11b
Merge
CD7
CD8
CD13
CD15
g
GZMB+ CD8+ T
GZMK-only
GZMK+ GZMB+
Lamina propria
Submucosa
Muscularis propria
h
Minimum distance from GZMK+ GZMB+ cells
Probability density
GZMK-only
81.90 ± 59.43 µm
GZMB+CD8+ T
89.31 ± 71.20 µm
Distance (µm)

Supplementary Fig. 7. a Spatial feature plots of marker genes for $GZMK^+CD8^+$ T cells and myeloid cells within the 10x Visium dataset. **b** Spatial feature plots highlighting colocalization of $GZMK^+CD8^+$ T cells with cDC2 in the highlighted region 3 of the 10x Visium dataset. **c** Receptor-ligand interaction scores incorporating spatial coordinates for CXCR3-CXCL10 and CXCR6-CXCL16 in the inflamed small intestinal tissue analyzed using 10x Visium. **d** Bar plot showing cell type counts for each cell in the inflamed small intestinal tissue analyzed by 10x Xenium. **e** Receptor–ligand interaction scores for CXCR3–CXCL10 and CXCR6-CXCL16 incorporating spatial coordinates in the 10x Xenium dataset. **f** Immunohistochemistry (IHC) images of inflamed small intestinal tissue from patients with CD stained for CD8, granzyme K, and CD11b. **g** Density plots depicting the spatial distribution patterns of GZMK-only, $GZMK^+GZMB^+$, and $GZMB^+CD8^+$ T cells in 10x Xenium spatial transcriptomics. **h** Spatial proximity of $GZMK^+GZMB^+$ cells to GZMK-only and $GZMB^+CD8^+$ T cells. Kernel density distributions of the minimum distance from GZMK-only (top) or $GZMB^+CD8^+$ T cells (bottom) to the nearest $GZMK^+GZMB^+$ cell. Dashed lines indicate mean distances, with mean ± SD shown for each distribution.

Supplementary Table 1. Clinical characteristics

Patient	Tissues	Modalities	Sex	Age at diagnosis	Location at diagnosis	Behavior at diagnosis	Disease duration (m)	Surgery	Cause of surgery	Treatment within 6 months of surgery	Smoking status at surgery	Sorted cells	Sorting methods
CD1	Uninflamed SI	scRNA-seq and scTCR-seq	M	21.9	ileocolonic	penetrating	1.0	Ileocolic resection	Intra-abdominal abscess	none	current	CD3+	MACS
CD2	Uninflamed SI	scRNA-seq and scTCR-seq	F	19.3	ileocolonic	stricturing	92.3	Ileocolic resection	Stricture	infliximab	never	CD3+	MACS
CD3	Uninflamed SI	scRNA-seq, scTCR-seq, and Immunohistochemistry	M	24.0	ileum	penetrating	0.8	Small bowel resection	Intra-abdominal abscess	steroids	current	CD3+	MACS
CD4	Uninflamed SI	scRNA-seq, scTCR-seq, and Immunohistochemistry	F	25.5	ileum	penetrating	188.9	Small bowel resection	Intra-abdominal abscess	azathiprine	ex	CD45+	MACS
CD5	Uninflamed SI	scRNA-seq and scTCR-seq	M	22.0	ileocolonic	stricturing	159.9	Subtotal colectomy	Stricture	azathiprine	never	CD45+	EasySep
CD6	Uninflamed SI	scRNA-seq and scTCR-seq	F	13.2	ileocolonic	NSNP	230.2	Right hemicolectomy	Intra-abdominal abscess	none	never	CD45+	EasySep
CD7	Uninflamed SI, Blood	scRNA-seq, scTCR-seq, and Immunohistochemistry	F	16.0	ileum	NSNP	115.7	Ileocolic resection	Intra-abdominal abscess	ustekimumab/azathiprine	never	CD3+	EasySep
CD8	Uninflamed SI	scRNA-seq, scTCR-seq, and Immunohistochemistry	M	22.4	ileum	NSNP	131.1	Ileocolic resection	Intra-abdominal abscess	azathiprine	never	CD3+	EasySep
CD9	Uninflamed SI, Blood	scRNA-seq and scTCR-seq	M	23.7	ileocolonic	unknown	97.4	Right hemicolectomy	Intra-abdominal abscess	ustekimumab/azathiprine	never	CD45+	EasySep
CD10	Inflamed SI, Uninflamed SI, Blood	scRNA-seq and scTCR-seq	F	39.9	ileocolonic	stricturing	97.1	Right hemicolectomy	Intra-abdominal abscess	azathiprine	never	CD45+	EasySep
CD11	Inflamed SI, Uninflamed SI, Blood	scRNA-seq and scTCR-seq	M	30.2	ileum	stricturing	89.0	Ileocolic resection	Stricture	none	current	CD45+	EasySep
CD12	Inflamed SI, Uninflamed SI, Blood	scRNA-seq and scTCR-seq	F	35.5	ileocolonic	unknown	57.0	Subtotal colectomy	Stricture	infliximab	ex	CD45+	EasySep
CD13	Inflamed SI, Uninflamed SI, Blood	scRNA-seq, scTCR-seq, 10x Xenium, and Immunohistochemistry	M	35.0	ileocolonic	penetrating	1.3	Small bowel resection	Intra-abdominal abscess	azathiprine	current	CD45+	EasySep
CD14	Inflamed SI, Uninflamed SI, Blood	scRNA-seq and scTCR-seq	M	18.1	ileocolonic	penetrating	59.9	Ileocolic resection	Stricture	adalimumab/azathiprine	current	CD45+	EasySep
CD15	Inflamed SI, Uninflamed SI, Blood	scRNA-seq, scTCR-seq, and Immunohistochemistry	M	20.3	ileum	penetrating	1.2	Small bowel resection	Intra-abdominal abscess	steroids/azathiprine	never	CD45+	EasySep
CD16	Inflamed SI	10x Visium	M	18.5	ileocolonic	unknown	147.0	Ileocolic resection	Intra-abdominal abscess	adalimumab/azathiprine	never	NA	NA
CD17	Uninflamed SI	Flow cytometry	F	23.1	ileocolonic	stricturing	126.2	Small bowel resection	Stricture	ustekimumab/azathiprine	never	NA	NA
CD18	Uninflamed SI	Flow cytometry	M	27.8	ileum	stricturing	148.6	Small bowel resection	Stricture	infliximab/ azathiprine	ex	NA	NA

NA: not applicable, NSNP: non-stricturing non-penetrating, scRNA-seq: single-cell RNA sequencing, scTCR-seq: single-cell T cell receptor sequencing, SI: small intestine.

Supplementary Table 2. Representative cell markers of 15 T/NK cell clusters

T/NK cell clusters		Marker genes
$CD8^+$ Trm	$CD8^+$ Tissue resident memory T cells	*CD8A, CD8B, XCL1, CD160, ITGAE, ITGA1, KLRC1, SPRY1, RUNX3, CXCR3*
$CD8^+$ Tcm	$CD8^+$ central memory T cells	*CD8A, CD8B, SELL, CCR7, KLF2, LEF1, TCF7*
$CD8^+$ Tem	$CD8^+$ effector memory T cells	*CD8A, CD8B, GZMB, GZMK, EOMES, FGFBP2, GZMH, GNLY, PRF1, IFNG, KLRD1, GZMA*
Th17 Trm	Tissue resident memory Th17 cells	*CD4, IL17A, IL22, CXCR6, CCL20, KLRB1, RORC, RORA, CCR6, IL23R, ITGAE, ITGA1*
Activated $CD4^+$	Activated $CD4^+$ T cells	*CD4, HLA-DRB1, HLA-DRA, TNF, HLA-DPA1, IFNG, HLA-DRB5, TGFB1, CXCR3*
$CD4^+$ Tem	$CD4^+$ effector memory T cells	*CD4, IL7R, ANXA1, LMNA, S100A11, VIM, TIMP1, ICOS*
$CD4^+$ Tcm	$CD4^+$ central memory T cells	*CD4, SELL, CCR7, KLF2, LEF1, TCF7, S1PR1*
$CD4^+$ Tfh	$CD4^+$ follicular helper T cells	*CD4, CXCR5, CXCL13, TOX2*
$CD4^+$ Treg	$CD4^+$ regulatory T cells	*CD4, FOXP3, CTLA4*
gd-T (1)	gamma delta T cells (1)	*TRDC, TRDV1, KLRC2, CD160, XCL1, GZMA*
gd-T (2)	gamma delta T cells (2)	*TRDC, TRDV2, CCL4, KLRG1, KLRD1, KLRC1*
gd-T (3)	gamma delta T cells (3)	*TRDC, GZMA, FCER1G, KIR2DL4, CD247, TYROBP*
gd-T (4)	gamma delta T cells (4)	*TRDC, ZNF683, XCL1, XCL2, TRDV1*
MAIT	Mucosal-associated invariant T	*TRAV1-2, NCR3, CCL20, CEBPD, ZBTB16*
NK cells	NK cells	*TYROBP, KLRF1, KLRC2, GZMB, GNLY, FCGR3A, FCER1G*

Supplementary Table 3. Gene signatures

**Effectorness (Figure 1D)** Cano-Gomez et al, 2020	**Cytotoxicity (Figure 1D)** Hashimoto et al, 2019	**Naïve (Figure 1D)** Abbas et al, 2009	**Residency (Figure 1D, Figure 2C)** Milner et al, 2017	**CD69+ Trm (Figure 2C)** Kumar et al, 2017	**Tissue gene (Figure 2C)** Szabo et al, 2019	**Jejunum Trm (Figure 2C)** Poon et al, 2023	**Core Trm (Figure 2C)** Poon et al, 2023	**Circulating (Figure 2C)** Milner et al, 2017	**Inflammation (Figure 4F)** Smillie et al, 2019
TNFRSF18	GZMA	AARS1	ADAM19	CA10	APOBEC3C	IL7R	VIM	ACSS1	IFNG
LMNA	GZMB	ABLIM1	ADGRG1	ITGA1	PERP	RGS1	LGALS3	AFF3	IFNGR1
GNLY	GZMH	ACER1	ATF3	ITGAE	FAM129A	KLRB1	SAMSN1	APOBEC2	IFNGR2
IQCG	PRF1	ACSS2	B4GALNT4	IL2	TUBA4A	JUND	SLC7A5	ARHGAP26	IL10
HOPX	NKG7	ACTN1	BAG3	IL10	OSTF1	KLF6	HMGB2	ARHGEF18	IL12A
GZMA	GNLY	ADGRA3	BHLHE40	CXCR6	ANXA2	TNFAIP3	EZR	ARMC7	IL12B
TMEM173	CD40LG	AEBP1	BTG2	CXCL13	S100A11	ATF3	ANKRD28	AS3MT	IL12RB1
HLA-DRA	KLRG1	AFF3	BTG3	KCNK5	PPP1R14B	ANXA1	SAP30	AVEN	IL12RB2
HLA-DRB1	KLRB1	AGMAT	CCL4	RGS1	CD63	ANKRD28	S100A11	B3GNT5	IL13
HLA-DQA1	ITGAL	AIF1	CD244	CRTAM	AP2B1	CCL5	LMNA	BIN2	IL17A
HLA-DPB1		AK5	CD69	DUSP6	RGCC	TSC22D3	RNF19A	CMAHP	IL17F
ACTB		ALS2CL	CHD7	PDCD1	PLEC	MYADM	COTL1	CYRIA	IL18
CTSW		AMN1	CISH	IL23R	HTATIP2	TMIGD2	ADAM19	DAPL1	IL18R1
C10orf128		APBA2	COQ10B		PRDX1	ZFP36	CDKN1A	DEAF1	IL18RAP
IFNG		ARMCX2	CREM		EMP3	UBC	PFKFB3	DENND2D	IL1A
GZMB		ATM	CTLA4		HNRNPH1	GPR171	ERN1	DGKA	IL1B
NFKBIA		BACH2	DENND4A		PRDX5	CD160	NR3C1	DOCK5	IL2
LGMN		BEND5	DGAT1		ITGA1	SPRY1	PDE4D	DUSP7	IL21
CCL5		BNIP3L	DNAJA1		KLF6	SMIM3	GADD45G	EHD3	IL21R
CCL4		CAMK4	DNAJA4		TAGLN2	LDLRAD4	RUNX2	ELOVL7	IL22
NKG7		CCDC110	DNAJB1		KRAS	CCNH	BTG3	EOMES	IL23A
EPAS1		CCDC3	DNAJB4		EZR	SAP30	RORA	F2RL2	IL23R
CSF2		CD55	DNAJB6		NR3C1	H1FX	AKAP13	FAM53B	IL2RG
CCL3		CD7	DUSP12		LGALS1	CKLF	IFNGR1	FGF13	IL4
		CD8B	DUSP4		LGALS3	ITGAE	CCR6	FLNA	IL4R
		CDCA7L	DUSP6		CD82	ITGA1	AHR	GAB3	IL5
		CENPV	EGR1		CD27	SESN1	PBX4	GRAMD4	IL6
		CEP41	EHD1		S100A10	PDE4D	RGS16	GSAP	JUN
		CERS6	ELL2		RP11-465B22.3	SAMSN1	PARD6G	GZMM	NFKB1
		CHI3L2	FASLG		AQP3	PPP1R15A	CPD	HAAO	RELA
		CHMP7	FBXW7		AMICA1	ODF2L	REEP3	ICAM2	RORA
		CHST2	FGL2		YWHAH	EZR	ELL2	IL17RA	RORC
		CLCN5	FMNL3		CRIP1	SLC7A5	LINC00892	IL18RAP	S100A8
		CNKSR2	FOS		JUN	SRSF7	ATXN1	KBTBD11	S100A9

CRLF3	FOSL2	VIM	AREG	SMIM3	KCNJ8	STAT1
CRTC3	FZR1	S100A6	VIM	TIPARP	KLF2	STAT3
DACT1	GABARAPL1	CAPG	TSPYL2	JMY	KLHDC1	STAT4
DBH-AS1	GADD45B	HMGB1P5	NR3C1	CRIM1	KLRG1	STAT6
DCAF16	GLRX	ANXA1	CD55	SYTL2	LAIR1	TGFB1
DDR1	GMNN	ITM2A	GFPT2	SMAD7	LFNG	TGFB2
DENND2D	GPR171	MRPS6	CCR9	C4orf32	LIMD1	TGFB3
DEPDC7	GZMB	ITM2C	CNOT10	VSTM2L	LPIN1	TNF
DNHD1	H3-3B	TMEM14C	AC092580.4	YWHAH	LY9	
DNMT3A	HAVCR2	TUBA1C	FAS	PDE4A	MBOAT1	
EDAR	HIP1	LPXN	RASGEF1B	RP11-138A9.2	NCK2	
EEF1G	HOMER2	PLP2	RORA	XCL1	NFATC3	
EFNA1	HPGDS	PHLDA1	MAP4	SETD7	NOD1	
ELFN2	HSP90AA1	CBX6	ADGRE5	NLRP3	NSG2	
ENOSF1	HSPA5	CLDND1	FOS	TMEM200A	PDE2A	
EPHA1	ICOS	TSC22D3	AHR	MLLT4	PRSS12	
FAM117B	IFNG	CARD19	UBB	MCAM	PRUNE1	
FBXL12	IFRD1	CTNNB1	ERN1	CTNNA1	PYCARD	
FCGRT	IL21R	SLC38A2	JUN	KIF5C	QPRT	
FCMR	IL4R	ITGAE	IL2	ITPRIPL1	RAP1GAP2	
FLNB	INPP4B	CEBPD	HSPH1	AUTS2	RASA3	
FOXP1	IRF4	TUBA1A	TAGLN2	TARS	RASGRP2	
GAL3ST4	ISG20	SLC3A2	ABCB1	IL2	RGS19	
GGT7	ISY1-RAB43	TUBA1B	GLIPR1	CYSTM1	RIPOR2	
GP5	ITGA1	TUBB4B	SYTL2	FAR2	S1PR1	
GPRASP1	JUN	TUBB	KIF13B	EPAS1	S1PR4	
GRAP	KDM6B	GLO1	PELO	TMEM64	SAMD3	
H1-0	LITAF	ANKRD28	DAPK2	FLJ20021	SELL	
HIPK2	LMNB1	STMN1	SH2D2A	GNAO1	SFXN3	
HIVEP1	LY6G5B	GGA2	IVNS1ABP	CD9	SH2D1A	
IGF1R	MPZ	SMIM3	AKAP13	HLF	SH3BP5	
IGIP	MXD1	PR3-476K8.4	FOSB	BMF	SIDT1	
IL6ST	MXI1		CEBPD	SLC6A6	SIKE1	
IPCEF1	NEURL1B		RP11-693N9.2	DUSP6	SLAMF6	
ITGA6	NFIL3		CAPG	SPAG1	SMPDL3B	
KBTBD11	NFKBIE		CCL20	SMCO4	ST3GAL1	
KLF7	NR4A1		PTPN22	ELOVL6	STARD10	
KLHDC1	NR4A2		ABI3	SDC4	TCF7	

KLHL13	*NR4A3*	*CD8A*	*RBBP8*	*TLR1*
KLHL2	*NTAN1*	*ERF*	*AFAP1*	*TMEM71*
KLHL3	*P2RY10*	*STIP1*	*C11orf84*	*TRAF3IP3*
KPNA5	*P4HB*	*PFKFB3*	*IL22*	*UBXN2B*
KRT18	*PDCD1*	*ELL2*	*XYLT1*	*USP33*
KYAT1	*PER1*	*PTGER4*	*GAB2*	*YPEL1*
LAGE3	*PLK3*	*MRPL18*	*MAML3*	*ZEB2*
LAMA5	*PLSCR1*	*SPON2*	*ARHGAP11A*	*ZNF367*
LEF1	*PMEPA1*	*GOLGB1*	*DHCR24*	
LEF1-AS1	*PPP1R15A*	*KDM6B*	*NEK10*	
LINC01089	*PRDX5*	*EPAS1*	*COL5A1*	
LINC01550	*PTP4A1*	*TIPARP*	*PVRL3*	
LRRN3	*QPCT*	*CRIP1*	*EMP1*	
LTBP3	*RBPJ*	*CPD*	*CDYL2*	
LZTS3	*RGS1*	*CHORDC1*	*VKORC1*	
MAML2	*RGS10*	*CLIC5*	*RP11-401F2.3*	
MANSC1	*RGS16*	*AUTS2*	*SLC4A8*	
ME3	*RHOB*	*GPR34*	*CD101*	
MEF2A	*RNASEH2A*	*SSBP4*	*RAPGEF5*	
MGA	*RNF149*	*HSP90AA1*	*KLF4*	
MLLT3	*RRAD*	*EPS15*	*CACNB3*	
MMAB	*SIK1*	*COTL1*	*CHRNA1*	
MPP1	*SKIL*	*RNF19A*	*PRKAR2B*	
MTHFD2L	*SLC16A6*	*TNFSF13B*		
MTMR9LP	*SLC25A21*	*IFNGR1*		
MYB	*SLC7A5*	*DSE*		
NBEA	*SPTY2D1*	*HIC1*		
NDFIP1	*SSBP2*	*IKZF3*		
NEMP1	*STK17B*	*THEMIS*		
NFKBIZ	*TGIF1*	*LZTFL1*		
NIPAL3	*TIGIT*	*HPGD*		
NOG	*TIPARP*	*CITED2*		
NPAS2	*TJP1*	*ITM2C*		
NUCB2	*TNF*	*HMGB2*		
NUDT12	*TNFAIP3*	*PTGER2*		
OBSCN-AS1	*TNFRSF1B*	*TPD52*		
PADI4	*TNFRSF9*	*CDKN1A*		
PCED1B	*TRA2A*	*MGAT5*		

PCSK5	*TRIB1*	*TLE1*
PDCD4-AS1	*UBE2S*	*LMNA*
PDE3B	*VPS37B*	*IER5*
PDE7A	*WSB1*	*SQSTM1*
PDE7B	*XCL1*	*HSPE1*
PDE9A	*ZFAND5*	*TMEM64*
PDK1	*ZFP36L1*	*ZFAND5*
PHGDH		*NLRP3*
PIGL		*SRGAP3*
PIK3CD		*DOK2*
PIK3IP1		*FAM177A1*
PITPNM2		*DNAJA4*
PLAC8		*HNRNPUL1*
PLAG1		*MAN1A1*
PLXDC1		*LINC00239*
PRICKLE2		*REL*
PRKCA		*JMY*
PRRT1		*MCAM*
PRXL2A		*LPIN2*
RAB3GAP1		*PMAIP1*
RAB43		*NFKBID*
RAPGEF6		*GABARAPL1*
RBM11		*ATF7IP*
RBM27		*NFKB2*
RGS10		*ODC1*
RIN3		*RP11-138A9.2*
RNF157-AS1		*MIR23A*
ROBO3		*NEDD9*
RPS5		*LIMS1*
SATB1		*RIPK2*
SCARB1		*CD83*
SCML2		*LAIR1*
SDHAP2		*MVB12B*
SERTAD2		*CA10*
SERTM1		*TRIM22*
SETD1B		*PBX4*
SFMBT2		*GPR65*
SFXN2		*NEU1*

SLC12A6
SLC16A10
SLC25A37
SLC8B1
SNHG32
SNHG7
SNN
SNPH
SPTBN1
SREBF1
TAF4B
TARBP1
TBC1D32
TBL1X
TCF7
TECPR1
TET3
TGFBR2
TMEM198B
TMEM245
TMEM272
TMEM41B
TOM1L2
TOP1MT
TPCN1
TRABD2A
TRMT13
TTC28
TTN
TUG1
UBE2B
UBE2E2
USP11
USP44
VNN2
ZBTB18
ZNF138
ZNF253

CMTM6
CA2
GYG1
IL18RAP
RHOB
HMGCS1
BTG3
BLMH
NBN
FSD1
MLF1
CENPM
HSPA6
TP53INP1
SLK
PLCB1
LGALS3
FOXO3
CRACR2A
FKBP4
CHD7
RELT
NBL1
MSH3
STOM
UBASH3B
KAT6B
ICAM1
FLJ20021
PARVB
HOPX
RUNX2
IL17A
GTDC1
CD58
PRR5
TMEM200A
ZFAND2A

ZNF275
ZNF43
ZNF439
ZNF496
ZNF506
ZNF510
ZNF516
ZNF563
ZNF708
ZNF763
ZNF780B

CLIC3
GPR55
CYSTM1
GCHFR
GALNT10
HSPD1
TSEN54
KIAA1109
NUDT4
DFNB31
HES4
TAB2
TIFA
S100A11
KIAA0513
SRI
AP3S1
YWHAH
ELOVL6
IL18R1
TRAF1
SMAD7
CCR6
PVRL3
SLC25A32
LINC00892
SNTA1
SLC7A5P1
RORC
GFI1
TPBG
SCRIB
AC131056.3
DZIP3
PLAT
OBFC1
ASB2
FAM3C

SERPINB9
ARMCX3
CD9
CD101
DNAJB4
DTX1
HOXB2
SLC4A7
KIT
ZDHHC14
PROK2
MIS18BP1
NUGGC
P4HA1
HSPA4
CRTAM
IFI44
RBM12
PNP
RBBP8
STARD4
ACSL1
MB21D1
NCF4
GAB3
CTD-3252C9.4
ERRFI1
NFIA
IL2RA
CD70
CCDC6
CRY1
XCL2
JAZF1
DYNC1LI1
HSPA1A
TRAF4
ARG2

PRKAR2B
MAP9
ZNF652
LINC00963
CAPN12
ADAM19
ARHGEF40
GFOD1
FAM122C
ATXN1
PDE4A
NRIP1
HCP5
KIAA1211L
C3AR1
ABHD15
PARD6G
KIF21A
C4orf32
SDC4
FURIN
GADD45G
COL5A1
PLAGL1
TNIP3
LAG3
CMIP
GNAO1
HMGCR
ARHGEF12
ME1
ABHD14A
GCNT1
TARS
MPZL3
PARD6A
LRRC1
HSPA13

CNDP2
NT5E
MAP4K5
KLRC1
BCAP29
TP53I13
MLLT4
CRIM1
SKAP2
RP11-325F22.2
IL12RB2
DOCK5
IFI44L
CXCR6
CLIP4
TBCD
RAB27A
SLC31A2
OSGIN2
SETD7
NPAS2
LDLR
WNT1
PLEKHF1
AC020571.3
IGFBP3
RNF14
KIF5C
CASP7
C11orf84
ENPP5
TNFSF9
ASAP1
EPOR
STXBP3
POC1B
TNFSF14
TFE3

SLC43A3
RHOQ
GCSAM
PQLC3
GPATCH2
TMEM71
C10orf128
ADCY3
ADAMTS17
GCLC
CREG1
ENTPD1
ANKRD9
XCL1
TMED8
CEACAM21
CNIH2
ABCA1
GAB2
RNF122
SQLE
CCDC47
EVC2
ERCC6L2
PHTF1
TBXAS1
SERPINH1
SLC19A2
CD151
BMF
IL4I1
OSBPL3
METTL14
BAIAP2
MAP1LC3A
LINC00665
PTPN13
SPG20

DHCR24
KLRC2
REEP3
IL15
SLC37A1
LRIF1
GNA15
P2RX4
ELL
FMNL3
CCDC117
AP000783.1
TRGV10
PNMA5
ZNF827
VAV2
XYLT1
SPEF2
RGS3
LRRN3
GPR18
SUV420H2
BBS7
PAQR8
RP11-38O23.4
MAP1A
KLF4
GOT1
GMPPB
RP11-162J8.3
CHSY1
MAGI3
TRGC2
ZBED4
TRPC3
SIK2
ATP8B4
RASGRF2

TRGV2
SLC25A4
LDOC1L
CD27-AS1
MPHOSPH9
RFX5
SLC6A6
PRSS35
DUSP6
ENPP4
FRMD4B
NR1D1
LAYN
QPCT
HDAC9
WDR86-AS1
C11orf63
UNG
IL17RE
PHLDA3
ZFY
AFAP1
CA3-AS1
PXYLP1
ITPRIPL1
COL5A3
CCDC50
SCMH1
IL22
CTNNA1
GBA2
SPAG1
H1F0
CSF1
CTBP1-AS
ARL5B
BRWD3
SMCO4

EEF1E1
CALY
MATN1-AS1
KLHL23
LIG4
LRRC41
SLC25A43
BZRAP1
DPY19L1
TGFBI
INPP5B
F8
RP11-305L7.3
LRRC2
ITGAV
FARSB
LINC01057
EXOC6
KATNAL1
FAM86C1
CYTH3
EPB41L5
CHN1
SLC44A3
CDKN2A
LRRCC1
PIANP
CRACR2B
NKILA
LMNB2
LINC00092
PLCG2
FAR2
TRGV5
GSE1
ASAH2B
MYO1E
NEK10

TANC2
SLC35A2
SMAD5
TBC1D16
RGS16
FOXJ1
BLZF1
NIPSNAP3A
RP11-37B2.1
LTK
DTWD1
MRPL12
RP11-61J19.5
NEK4
GALNT7
CHRNA1
KIR2DL4
TSC22D1
IGSF22
ACOT7
CMC4
GPD1L
CNNM4
FSCN1
LINC00539
SLC7A8
MAML3
RP11-401F2.3
ATG7
FAM167A
FADS2
CACNB3
ZFP92
SLC4A10
TEC
CFAP54
TTC30B
PHKG2

RP11-245D16.4
DENND3
CXXC5
NMUR1
SLC22A18
RETSAT
SRXN1
KDELC1
NPM2
ENDOD1
WDR63
SEMA6C
ATP1B2
EVC
EGR4
BLOC1S5-1
NIPA1
TRMT11
TMCC3
PLEKHG1
WDR86
SLC44A1
RAPGEF5
TRIP6
SLC29A1
FADS1
C17orf107
SMC2
FEN1
RP11-384K6.6
HIST1H4D
VKORC1
SLC25A15
KLRC4-KLRK1
USP45
C11orf96
RP11-412D9.4
TULP3

PHLDB1
FAH
RP5-1180D12.1
IFIT3
IFT140
RP11-236P24.3
MMP24
UNQ6494
PPP1R13L
MPZL2
SPRY2
ZNF512
MYO1C
HLF
CDYL2
SLC6A8
IGHV1-3
ZNF280C
VSTM2L
RP1-234P15.4
CLECL1
RAD18
AHRR
PLCB3
PCAT6
ZNF597
IL23R
PWWP2B
WASF1
CLN6
EMP1
RP11-452L6.5
TRIM17
CLNK
SLC4A8
ST7-AS1
RP11-244H3.4
GSG2

IL26
C11orf72
CENPJ
KLHL26
PSEN2
LL22NC03-75H12.2
ZNF354C
ADAM12
RP11-126O1.6
ARHGAP11A
GRAMD1C

Supplementary Table 4. 10x Xenium Gene List

Gene list																
IL6	*TPM2*	*PIM1*	*SERPINB3*	*CXCL6*	*PRDX2*	*EIF4EBP1*	*CCNE1*	*CTSD*	*IL10*	*MSR1*	*HLA-A*	*GZMB*	*ITGAE*	*RHOH*	*SARAF*	*POU2AF1*
IL11	*MMP11*	*MMP12*	*SEMA3C*	*SERPINA3*	*DMKN*	*RHEB*	*CCR2*	*CX3CR1*	*IL1A*	*PDGFA*	*ENTPD1*	*TRDC*	*KLRK1*	*RORC*	*TNFRSF18*	*PAX5*
CCL2	*HOPX*	*MMP2*	*DST*	*DUOX1*	*ANXA1*	*GSK3B*	*CCR5*	*CXCL10*	*IL1B*	*PTK2*	*ICOSLG*	*GZMK*	*NELL2*	*SELL*	*ADORA2A*	*BTLA*
CCL22	*THBS2*	*SFRP4*	*EGFR*	*NTRK2*	*GPX2*	*WNT1*	*CCR7*	*CXCL11*	*IL23A*	*S100A12*	*ICAM1*	*ITGB1*	*SPON2*	*SMAP2*	*BTN3A1*	*XBP1*
CLEC3B	*LRRC15*	*VWF*	*MYC*	*KRT13*	*CDKN1A*	*AXIN1*	*CD14*	*CXCL9*	*IL4R*	*SIGLEC10*	*TLR4*	*TCF7*	*TNFRSF1B*	*TENT5C*	*CD27*	*IL12A*
COL14A1	*MYLK*	*WARS*	*NFKB1*	*KRT4*	*CCND1*	*APC*	*CD163*	*CXCR4*	*INHBA*	*SORL1*	*ITGB2*	*LEF1*	*TNFRSF9*	*TUBA4A*	*CD70*	*FGFR2*
LY6G6C	*TNC*	*SOX17*	*FAS*	*APOC1*	*TGFBR1*	*CTNNB1*	*CD1C*	*DUSP2*	*ISG15*	*SPP1*	*CD3E*	*IL32*	*TNFSF9*	*CD2*	*IL2*	*FGFR3*
CCL17	*COL1A2*	*STC1*	*ABCC11*	*ARG1*	*STK4*	*TCF7L1*	*CD1E*	*E2F3*	*ITGAV*	*TNF*	*CD3D*	*RTKN2*	*TRAT1*	*ITM2A*	*IL13*	*FGFR4*
CXCL1	*COL8A1*	*TAC1*	*CEACAM6*	*SPINK1*	*STK3*	*TP53*	*CD247*	*EDN1*	*ITM2C*	*TNFAIP6*	*IL7R*	*FOXP3*	*TSPAN32*	*S100A4*	*SELP*	*TERT*
CXCL2	*COL15A1*	*TCEAL7*	*CENPF*	*TFF3*	*SAV1*	*CHEK2*	*CD274*	*EGF*	*JAG1*	*TNFSF12*	*LTB*	*IL2RA*	*XCL1*	*IFITM1*	*SLAMF7*	*NKX2-1*
CXCL12	*PDPN*	*CCDC80*	*GATA3*	*KRT19*	*LATS1*	*MDM2*	*CD276*	*EPHB1*	*KIT*	*TNFSF8*	*CD4*	*TIGIT*	*ZNF683*	*TRBC2*	*IL4*	*BRD4*
CXCL14	*FBLN1*	*AKR1C3*	*KRT7*	*AR*	*LATS2*	*ATM*	*CD40*	*EZH2*	*KYNU*	*TPSAB1*	*CD8A*	*CTLA4*	*NCAM1*	*FGFBP2*	*MS4A1*	*PAK1*
LIF	*PDGFRA*	*AQP1*	*MKI67*	*CAV1*	*MAX*	*APOBEC3A*	*CD44*	*FABP5*	*LAMP3*	*TREM1*	*LDHB*	*FCRL3*	*CEACAM8*	*SOX4*	*FCRL1*	*ROS1*
HGF	*SERPINF1*	*IGF1*	*TOP2A*	*CDH1*	*MGA*	*C1QA*	*CD47*	*FASLG*	*LGMN*	*TREM2*	*TPT1*	*IKZF2*	*CD3G*	*TC2N*	*IGHM*	*CDK8*
ACTA2	*HLA-DRA*	*LUM*	*PCLAF*	*ERBB3*	*NOTCH1*	*C1QB*	*CD68*	*FCER1A*	*LILRA4*	*TYMP*	*TRAC*	*NOSIP*	*CD69*	*AQP3*	*COCH*	*ERCC2*
TGFB1	*SYTL1*	*PECAM1*	*TACSTD2*	*FLNB*	*HES1*	*C1QC*	*CD86*	*FCGR3A*	*LILRA5*	*VAV2*	*CTSW*	*CD8B*	*CYTIP*	*ITGA4*	*AIM2*	*ETV1*
POSTN	*AIFM2*	*TIFA*	*ZEB1*	*CTTN*	*NOTCH2*	*CCL13*	*CLEC10A*	*FCGR3B*	*LILRB2*	*VCAN*	*NKG7*	*ICOS*	*GPR183*	*TMSB10*	*BANK1*	*FOXA1*
TAGLN	*PDGFRB*	*KRT18*	*ZEB2*	*TPD52*	*HEY1*	*CCL18*	*CLEC4E*	*FGF18*	*LYVE1*	*VEGFA*	*KLRD1*	*CD40LG*	*GZMA*	*HCST*	*SSPN*	*MET*
THY1	*FGF1*	*ADIPOQ*	*SLC39A6*	*GPRC5A*	*PDK1*	*CCL20*	*CLEC7A*	*FGFR1*	*MERTK*	*VSIG4*	*TYROBP*	*CXCR6*	*HAVCR2*	*CD38*	*CD79A*	*MITF*
CCN2	*CLIC3*	*EDNRB*	*KRT14*	*ELF3*	*AKT1*	*CCL24*	*CLEC9A*	*FN1*	*MMP14*	*VTCN1*	*GNLY*	*DGKA*	*KLRB1*	*TOX2*	*TEX9*	*HRAS*
IGFBP3	*ANGPT1*	*MICA*	*KRT8*	*CLDN4*	*PTEN*	*CCL4*	*CPA3*	*FOLR2*	*MMP19*	*WDFY4*	*FCER1G*	*FHIT*	*KLRC1*	*NCR1*	*TNFRSF13C*	*ALK*
FAP	*VIM*	*EPCAM*	*MUC6*	*AGR2*	*PIK3CA*	*CCL5*	*CSF1*	*FYN*	*MMP9*	*WNT7B*	*PRF1*	*GZMH*	*LAG3*	*PTPRC*	*IGHA2*	*FLT3*
MYL9	*APCDD1*	*ERBB2*	*MYBPC1*	*TMC5*	*TSC1*	*CCL8*	*CSF3R*	*HPGDS*	*MRC1*	*CD80*	*KLRF1*	*IFNG*	*PDCD1*	*CXCL13*	*TNFRSF17*	*RHOA*
TPM1	*DPT*	*FHL2*	*PDCD1LG2*	*S100A6*	*TSC2*	*CCND2*	*CTSC*	*IDO1*	*MS4A6A*	*ELANE*	*CST7*	*IL2RB*	*PRDM1*	*SPOCK2*	*TNFRSF13B*	*IKBKE*
CCL21	*EOMES*	*FCER2*	*TP63*	*CLDN6*	*FOLR1*	*ITGB6*	*MTOR*	*TLR9*	*APOE*	*CTSS*	*G0S2*	*SOD2*	*THBS1*	*S100A9*	*S100A8*	*CXCL3*
CXCR2	*SIRPA*	*VSIR*	*CD96*	*ERF*	*ALDH1L1*	*CXCL5*	*EREG*	*PROX1*	*FASN*	*WT1*	*ADH1B*	*CXCL16*	*ITGA1*	*MMP3*	*DCN*	*MCAM*
CARD11	*MAPK1*	*AGO2*	*AXL*	*SRC*	*BCL6*	*AURKA*	*JUN*	*BRAF*	*GLI1*	*KDR*	*FGF19*	*RAC1*	*DNMT3B*	*KRAS*	*RAF1*	*NTRK1*
MS4A2	*IGHD*	*IL17RB*	*RB1*	*IL17A*	*ITK*	*PTGDS*	*VEGFD*	*CCL19*	*DDR2*	*RUNX1*	*BCL2*	*STAT3*	*SOX2*	*NOX4*	*CSF1R*	

Supplementary Table 5. Histological scoring for DSS-induced colitis across experimental replicates.

**Group**	**Name**	**Experimental batch**	**mononuclear cell infiltrate (0, absent; 1, mild; 2, moderate; 3, severe)**	**crypt hyperplasia (0, absent; 1, mild; 2, moderate; 3, severe)**	**epithelial injury/erosion (0, absent; 1, mild; 2, moderate; 3, severe)**	**polymorphonuclear cell infiltrates (0, absent; 1, mild; 2, moderate; 3, severe)**	**transmural inflammation (0, absent; 1, submucosal; 2, one focus extending into muscularis and serosa; 3 up to five foci extending into muscularis and serosa; 4, diffuse)**	**Total**
Ctrl	**#A-1**	Replicate 1	0	0	0	0	0	0
Ctrl	**#A-2**	Replicate 1	0	0	0	0	0	0
Ctrl	**#A-3**	Replicate 1	0	0	0	0	0	0
Ctrl	**#A-4**	Replicate 2	0	0	0	0	0	0
Ctrl	**#A-5**	Replicate 2	0	0	0	0	0	0
Ctrl	**#A-6**	Replicate 2	1	0	0	0	0	1
Ctrl	**#A-7**	Replicate 2	1	0	0	0	0	1
Ctrl	**#A-8**	Replicate 2	0	0	0	0	0	0
DSS + PBS	**#B-1**	Replicate 1	1	3	3	2	1	10
DSS + PBS	**#B-2**	Replicate 1	2	3	3	3	1	12
DSS + PBS	**#B-3**	Replicate 2	3	3	3	2	2	13
DSS + PBS	**#B-4**	Replicate 2	2	3	2	2	2	11
DSS + PBS	**#B-5**	Replicate 2	3	3	2	3	1	12
DSS + PBS	**#B-6**	Replicate 2	3	3	3	2	3	14
DSS + GZMK-inhibitor	**#C-1**	Replicate 1	2	2	2	2	1	9
DSS + GZMK-inhibitor	**#C-2**	Replicate 1	2	2	1	2	1	8
DSS + GZMK-inhibitor	**#C-3**	Replicate 1	1	1	2	2	1	7
DSS + GZMK-inhibitor	**#C-4**	Replicate 2	3	2	2	3	1	11
DSS + GZMK-inhibitor	**#C-5**	Replicate 2	2	3	2	2	1	10
DSS + GZMK-inhibitor	**#C-6**	Replicate 2	2	3	1	1	2	9
DSS + GZMK-inhibitor	**#C-7**	Replicate 2	3	3	2	2	1	11
DSS + GZMK-inhibitor	**#C-8**	Replicate 2	2	2	2	2	1	9
